# Structures of Cancer Antigen Mesothelin and Its Complexes with Therapeutic Antibodies

**DOI:** 10.1158/2767-9764.CRC-22-0306

**Published:** 2023-02-01

**Authors:** Jingyu Zhan, Dong Lin, Nathan Watson, Lothar Esser, Wai Kwan Tang, Alex Zhang, Xiufen Liu, Raffit Hassan, Anne Gleinich, Asif Shajahan, Parastoo Azadi, Ira Pastan, Di Xia

**Affiliations:** 1 Laboratory of Cell Biology, Center for Cancer Research, NCI, Bethesda, Maryland.; 2 Laboratory of Molecular Biology, Center for Cancer Research, NCI, Bethesda, Maryland.; 3 Thoracic and GI Malignancies Branch, Center for Cancer Research, NCI, NIH, Bethesda, Maryland.; 4 Complex Carbohydrate Research Center, University of Georgia, Athens, Georgia.

## Abstract

The tumor-associated antigen mesothelin is expressed at high levels on the cell surface of many human cancers, while its expression in normal tissues is limited. The binding of mesothelin to the tumor-associated cancer antigen 125 (CA-125) can lead to heterotypic cell adhesion and tumor metastasis within the pleural and peritoneal cavities. Immunotherapeutic strategies targeting mesothelin are being intensively investigated. Here, we report the crystal structures of mesothelin that reveal a compact, right-handed solenoid consisting of 24 short helices and connecting loops. These helices form a nine-layered spiral coil that resembles ARM/HEAT family proteins. Glycan attachments have been identified in the structure for all three predicted N-glycosylation sites and confirmed with samples from cell culture and patient ascites. The structures of full-length mesothelin and its complex with the Fab of MORAb-009 reveal the interaction of the antibody with the complete epitope, which has not been reported previously. The N-terminal half of mesothelin is conformationally rigid, suitable for eliciting specific antibodies, whereas its C-terminal portion is more flexible. The structure of the C-terminal shedding-resistant fragment of mesothelin complexed with a mAb 15B6 displays an extended linear epitope and helps explain the protection afforded by the antibody for the shedding sites.

**Significance:**

The structures of full-length mesothelin and its complexes with antibodies reported here are the first to be determined experimentally, providing atomic models for structural organization of this protein and its interactions with antibodies. It offers insights into the function of mesothelin and guidance for further development of therapeutic antibodies.

## Introduction

Mesothelin (MSLN) was discovered as a cell surface protein normally expressed by mesothelial cells lining the pleura, pericardium, and peritoneum (1, 2). It is aberrantly expressed in a variety of cancers including mesothelioma, and ovarian, pancreatic and lung cancers (3–8). Patients with high MSLN expression levels on their cancers often have a poor prognosis (9). Because MSLN is highly expressed on the surface of many cancers, it is now considered an excellent target for antibody-based immunotherapies (9–15). Although the physiologic function of MSLN remains unclear, studies have shown that it is capable of binding to the tumor-associated cancer antigen 125 (CA-125), also known as MUC16, mediating cell adhesions (16–19). By truncation and alanine replacement mutagenesis, the CA-125 binding site was mapped to a 64-residue fragment at the N-terminus of MSLN (18). MSLN has also been shown, under stressed conditions, to form a signaling complex with CA-125 and Thy1, thymus differentiation antigen 1, to regulate fibrogenic activation and proliferation of activated portal fibroblasts (20).

**FIGURE 1:**
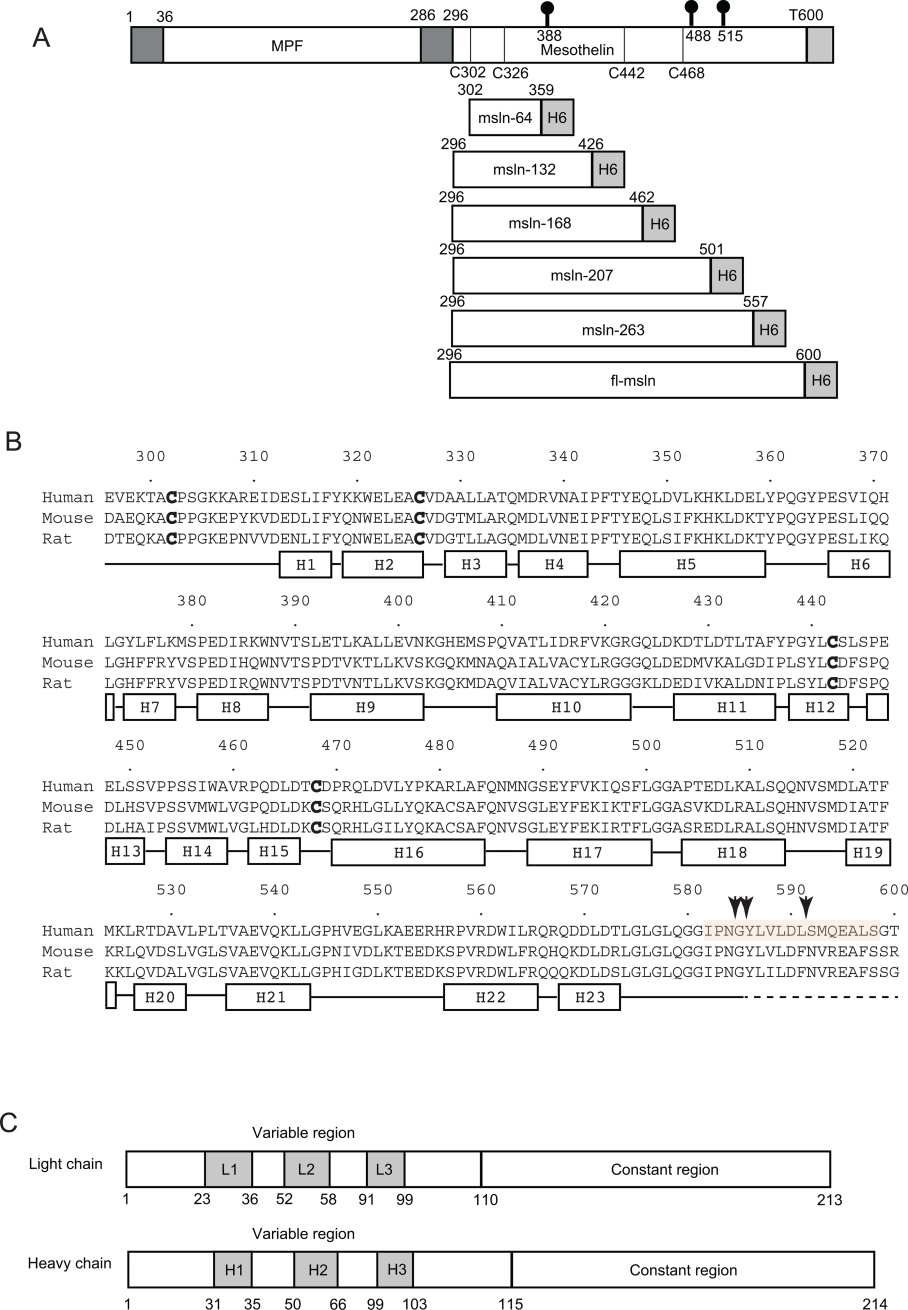
MSLN constructs, structure-based sequence alignment, and schematics of mAb 15B6. **A,** The top panel shows the precursor protein of the major isoform (Q13421-3) encoded by the human *MSLN* gene. The 622-residue precursor was subsequently processed by the endoprotease furin in the trans Golgi apparatus to form mature MSLN containing residues from E296 to T600. The three predicted N-glycosylation sites (N388, N488, and N515) are indicated. The conserved C302/C326 pair and C442/C468 pair form disulfide bridges, as determined by the crystal structures. In addition to the previously reported fl-MSLN and MSLN-64 (24), four more constructs with a C-terminal hexahistidine tag (H6) each were designed and named MSLN-132, MSLN-168, MSLN-207, and MSLN-263, respectively. **B,** Sequence alignment of MSLN from human, mouse, and rat genes. The conserved C302/C326 and C442/C468 pairs that form disulfide bridges are highlighted in boldface. Residues that form helices in the structure are shown as rectangles below the sequence alignment. Residues that form loops connecting helices are shown as black lines. The dashed line at the C-terminus indicates a disordered region. The peptide formed by residues from I582 to S598 used to elicit mAb 15B6 is highlighted and the major cleavage sites by membrane-bound sheddases are indicated by the black arrows. **C,** Schematics of the Fab light and heavy chains are shown for mAb 15B6. Residue numbering is consistent with prior reports and the CDRs in shaded boxes are assigned according to the improved Chothia method. CDRs are labeled as L1, L2, and L3 for the light chain and H1, H2, and H3 for the heavy chain. The variable and constant domains are indicated.

The gene that encodes human mesothelin (MSLN gene) is located on chromosome 16 (16p13.3) and produces a 68-kDa precursor protein that is subsequently processed by the endoprotease furin in the trans Golgi apparatus to yield a 40 kDa glycosylphosphatidylinositol (GPI)-anchored mature MSLN (2) and a 31 kDa megakaryocyte-potentiating factor (MPF; ref. 21; Fig. 1A). MSLN has three predicted N-glycosylation sites at positions N388, N488, and N515. Interaction with CA-125 was indicated to be sensitive to MSLN's glycosylation states (16). Interestingly, chimeric antigen receptor (CAR)-T cells generated on the basis of the N-terminal region (296–390) or the glycosylated C-terminal region (487–598) demonstrated differential killing of tumor cells, suggesting, among various factors, a role in MSLN's glycosylation states in T-cell recognition (22).

MSLN shares sequence homology with stereocilin and otoancorin; these proteins are also GPI anchored to the membrane of the inner ear sensory and nonsensory epithelial cells and are associated with deafness in people. It was predicted to have a superhelical structure with armadillo (ARM)-type repeats (23). Experimental structural information on MSLN had only been available for its N-terminal 64-residue fragment in complex with a therapeutic mAb MORAb-009 (PDB:4F3F; ref. 24). Among various mAb tested such as MN and MB (25), MORAb-009 was found to be a promising mAb with potential clinical applications (26–29) that went beyond its direct binding to MSLN. Its Fv fragment was tested as a carrier to deliver various anticancer agents to target cells. An anti-MSLN recombinant immunotoxin, SS1(dsFv) PE38 or SS1P, composed of the Fv portion of MORAb-009 (SS1) and a truncated form of pseudomonas exotoxin (30), was developed and evaluated in clinical studies (5, 14).

Recent studies have also shown that the efficacy of antibodies against MSLN is modulated by its shedding from cells due to the action of membrane-bound sheddases that cleave MSLN at various sites close to the cell membrane (31–33). Shedding releases antibody-based therapeutic agents from the cell surface before they can take effect; shed MSLN accumulating in the blood, ascites and pleural fluid can act as a decoy, preventing antibody-based agents from reaching the target cancer cells. Compounding this problem is the fact that various members of the ADAM (a disintegrin and metalloprotease), MMP (matrix metalloprotease), and BACE (β-secretase) family proteases were shown to take part in the shedding process in different cells. To overcome this problem, mAb 15B6 was developed; it targets a C-terminal fragment of MSLN, covering all the major protease cleavage sites. This mAb is able to block MSLN shedding and does not bind to shed MSLN. In addition, it causes complete remission of MSLN-expressing tumors in a mouse model when used in CAR-T cells (34).

Despite all the progress made, information on the structure of full-length MSLN (fl-MSLN) and its conformational variability are lacking. Here, we report the crystal structures of a 207-residue MSLN fragment, of fl-MSLN alone and in complex with a therapeutic mAb MORAb-009, as well as the structure of its C-terminal shed-resistant peptide complexed with mAb 15B6. We also analyzed glycan structures of MSLN from recombinant proteins, shed from cultured cells, and from patient ascites. Our findings revealed the structural similarity of MSLN to ARM/HEAT family proteins, which suggested its potential cellular functions, and shed new light on the conformational variability of MSLN. The complete structural information of MSLN could also guide the design and development of future MSLN-targeting cancer treatments.

## Materials and Methods

### Expression and Purification of the Full-length Wild-type MSLN

Full-length cDNA of MSLN was inserted into the baculovirus transfer vector pAcGP67A (RRID:Addgene_41812) of BD BaculoGold (BD Biosciences) in frame with the hexahistidine tag at the C-terminus (Supplementary Table S1). All mutations were made by PCR using the QuikChange mutagenesis kit (Agilent Technologies, Inc.). The plasmid was cotransfected with linearized viral DNA into approximately 2 million Sf9 cells and the culture was gradually amplified to 10 L of cultured insect cells for secretory expression of MSLN. Culture media were collected and concentrated in a diafiltration device (Millipore) against a diafiltration solution containing 25 mmol/L Tris, pH 7.5, 300 mmol/L NaCl, and 10% glycerol. The sample was then mixed with Ni-NTA resin (Qiagen) preequilibrated with the same buffer supplemented with 10 mmol/L imidazole. After washing with the diafiltration buffer supplemented with 50 mmol/L imidazole, bound MSLN was eluted in the presence of 100 mmol/L imidazole. Fractions containing MSLN were pooled and concentrated. MSLN was further purified by size-exclusion chromatography (SEC) using a Superdex 75 column equilibrated with 20 mmol/L Tris, pH 8.0, 100 mmol/L NaCl. Fractions were pooled, concentrated to 16 mg/mL using an Amicon Ultra concentrator (Millipore) with MWCO 30 kDa and stored at −80°C until use.

### Expression, Refolding, and Purification of Various MSLN Constructs

The *Escherichia coli* (*E. coli*) strain Rosetta (DE3) harboring the plasmid pET24a-MSLN-nn (nn = 132, 168, 207, or 263; Supplementary Table S1) was cultured in 1 L LB medium at 37°C, supplemented with 50 μg/mL kanamycin and 34 μg/mL chloramphenicol. The culture was allowed to grow to an OD_600_ of 0.8 before expression of recombinant proteins was induced at 37°C for 3 hours by adding isopropyl-β-D-1-thiogalactopyranoside to a concentration of 0.5 mmol/L. Cells were pelleted down by centrifugation and lysed by sonication in 80 mL lysis buffer (20 mmol/L Tris, pH 8.0, 0.5 mol/L NaCl, and 1 mmol/L phenylmethylsulfonyl fluoride). The cell lysate was centrifuged at 38,729 × *g* for 20 minutes. For MSLN-132 that was found soluble in the supernatant, the supernatant was then mixed with Ni-NTA resin (Qiagen) preequilibrated with washing buffer A (25 mmol/L Tris, pH 7.5, 150 mmol/L NaCl, 10% glycerol, and 20 mmol/L imidazole adjusted to pH 7.5) and incubated for 2 hours at 4°C. The resin was then transferred to a column. After washing the column with the washing buffer A, the bound protein was eluted by the same buffer supplemented with 300 mmol/L imidazole. The eluant was concentrated and further purified by SEC on a Superdex S-200 column equilibrated with a SEC running buffer (20 mmol/L Tris, pH 8.0, 100 mmol/L NaCl, 1 mmol/L β-ME, and 2% glycerol). The fractions containing the MSLN fragment were pooled and stored at 4°C for later use.

For MSLN-168, MSLN-207, and MSLN-263, which were found to be present in the form of inclusion bodies in the pellet, the pellet was washed in 80 mL of washing buffer B (2 mol/L urea, 50 mmol/L Tris, pH 8.0, 0.5 mol/L NaCl, 5% Glycerol, 1% triton X-100, 10 mmol/L β-ME, and 1 mmol/L Ethylenediaminetetraacetic acid (EDTA)), sonicated for 5 minutes and then centrifuged at 38,729 × *g* for 20 minutes. This washing step was repeated two more times. The purified inclusion bodies were dissolved in 80 mL solubilization buffer (8 mol/L urea, 50 mmol/L Tris, pH 8.0, 0.5 mol/L NaCl, 5% glycerol, and 10 mmol/L β-ME). The solution was stirred for 1 hour at 4°C and centrifuged at 38,729 × *g* for 20 minutes to remove any insoluble material. The supernatant was applied to an 8-mL Ni-NTA column preequilibrated with an equilibration buffer (6 mol/L urea, 50 mmol/L Tris, pH 8.0, 0.5 mol/L NaCl, 5% glycerol, 10 mmol/L β-ME, and 10 mmol/L imidazole), which was washed with 2.5 column volume of the equilibration buffer. The bound protein was eluted with 20 mL elution buffer (4 mol/L urea, 50 mmol/L Tris, pH 8.0, 0.5 mol/L NaCl, 5% glycerol, 10 mmol/L β-ME, 300 mmol/L imidazole). The eluate was concentrated to 2–4 mg/mL using an Amicon Ultra concentrator MWCO 10 kDa (Millipore) and further purified by SEC on a Superdex S-200 column equilibrated with the SEC running buffer (20 mmol/L Tris, pH 8.0, 100 mmol/L NaCl, and 1 mmol/L β-ME). The fractions containing refolded protein were pooled, concentrated to 10 mg/mL and stored at −80°C for later use.

### Preparation of Fab Fragment of IgG

The Fab fragment of the MSLN-specific mAb MORAb-009 (Morphotek Inc), Fab(MORAb), was prepared following the instructions provided by the Fab preparation kit (Thermo Fisher Scientific) as described previously (24). Briefly, 0.5 mL of the MORAb-009 at 5 mg/mL was first run through a desalting column preequilibrated with the supplied digestion buffer and was digested by mixing with 0.125 mL of resin-immobilized papain at 37°C for 4 hours. Fc fragments and undigested IgG were removed by incubating with a Protein A column and the Fab fragments were eluted with PBS buffer. Purified Fab from 4 mL of IgG were pooled and concentrated to 8 mg/mL using an Amicon Ultra concentrator (Millipore) with MWCO of 30 kDa.

The mAb 15B6 targeting the C-terminal peptide of MSLN was obtained from GenScript. The Fab fragment of 15B6, Fab(15B6), was prepared similarly as for MORAb-009. Briefly, mAb 15B6 at 5.6 mg/mL was buffer exchanged into the supplied digestion buffer using a Zeba Spin Desalting Column. The digestion was carried out by mixing 15B6 with preequilibrated immobilized papain and incubating at 37°C with constant mixing. After 4 hours digestion, the reaction was stopped by separating the digest from the immobilized papain using centrifugation. The digested Fab/Fc mixture was passed through a NAb Protein A Plus spin column to remove Fc and any undigested IgG, and the Fab fragment in the flow through was washed out of the column with TBS buffer containing 20 mmol/L Tris and 100 mmol/L NaCl at pH 7.5. Purified Fab was buffer exchanged into TBS and concentrated to 9.4 mg/mL using an Amicon Ultra 30 kDa centrifugation filter (Millipore). The Fab digestion and purification process were monitored by SDS-PAGE.

### Blue-native PAGE Analysis of Mesothelin-Fab(MORAb) Complex

The purified fl-MSLN in 25 mmol/L Tris, pH 7.5, 200 mmol/L NaCl was mixed with MORAb-009 Fab and incubated at room temperature for 30 minutes to allow formation of the complex. The complex was then subjected to blue-native PAGE (BN-PAGE) analysis following the procedure described previously (35).

### Formation and Purification of fl-MSLN/Fab(MORAb) Complex

To prepare the complex of fl-MSLN with Fab(MORAb), purified Fab(MORAb) (8 mg/mL or 0.16 mmol/L) in PBS was mixed with an excess amount of MSLN in a 1:1.1 molar ratio in a buffer consisting of 20 mmol/L Tris, pH 8.0, 100 mmol/L NaCl, 1 mmol/L β-ME, and 2% glycerol and the resulting mixture was incubated at 4°C overnight. Excess fl-MSLN and impurities were removed using a Superdex S-200 column with a running buffer consisting of 20 mmol/L Tris, pH 8.0, 100 mmol/L NaCl, 2% glycerol. Each eluted fraction was checked with SDS-PAGE and those containing the complex were pooled and concentrated to 6 mg/mL for crystallization using an Amicon Ultra MWCO 30 kDa centrifugation filter (Millipore).

### Crystallization of MSLN Variants

Crystallization of the fl-MSLN was performed robotically using a Mosquito liquid dispenser (TTP LabTech) in a 96-well hanging-drop format (Greiner 96-well U-shape plate ref: 650101). For initial screening, 85 μL of reservoir solutions from a commercial screen kit Index HT (Hampton Research) was used, and the drops were set up with 240 nL of protein at 10 mg/mL in 50 mmol/L Tris (pH 7.5) and 200 mmol/L NaCl with 240 nL of the reservoir solution. The crystallization hit condition (B6) obtained from the screen was refined. The final conditions for crystallization were mix of the protein in a 1:1 ratio with a well solution containing 0.9–1.1 mol/L Na/K phosphate, pH 8.2.

Crystallization of MSLN-207 was done similarly. The protein had a concentration of 4.0 mg/mL in a solution containing 20 mmol/L Tris, pH 8, 100 mmol/L NaCl, and 1 mmol/L β-ME. The well solution had a volume of 60 μL containing 0.1 mol/L HEPES pH 7.5, 100 mmol/L KCl, 12.5% PEG3350, 100 mmol/L Li_2_SO_4_. Protein and well solution were mixed in a 1:1 ratio (0.3 μL each) and incubated at 16°C. Crystals appeared after several months. Before diffraction data collection, crystals were treated with a cryoprotectant solution made of 100% glycerol and the well solution in a 1:2 ratio.

The crystallization of fl-MSLN/Fab of MORAb-009 complex was also performed robotically by the hanging-drop method. An aliquot of 0.3 μL protein of fl-MSLN/Fab(MORAb) complex at 6 mg/mL concentration in a buffer containing 25 mmol/L Tris, pH 7.5, 150 mmol/L NaCl, 2% glycerol was mixed with 0.3 μL reservoir solution (60 μL) containing 0.1 M Tris, pH 7, 150 mmol/L Na-citrate, 16.4% PEG3350, and 100 mmol/L Li_2_SO_4_. Crystals appeared within one week to several months. Crystals were treated with a cryoprotectant solution made of 100% glycerol and reservoir solution in a 1:2 ratio.

The first step in crystallizing the C-terminal peptide (residues 582–598)/Fab of Mab 15B6 complex was to dissolve the synthetic peptide powder (GenScript) in the same TBS buffer (20 mmol/L Tris and 100 mmol/L NaCl, pH 7.5) as the Fab(15B6). The peptide solution at 4.4 mg/mL was then mixed with concentrated Fab(15B6) (9.4 mg/mL in 20 mmol/L Tris and 100 mmol/L NaCl, pH 7.5) at a molar ratio of 3:1 and left on a horizontal orbital shaker at 4°C overnight before setting up the crystallization trays. Crystallization was carried out robotically in a 96-well format. The reservoir solution (85 μL) contained 0.1 mol/L Na acetate, pH 4.5, 26% PEG 550 MME, 6% 2-propanol. Hanging drops were formed with 240 nL reservoir and 240 nL protein and incubated at 4°C. Small crystals appeared after one day and matured within 1 week through an Ostwald-ripening process. Crystals were flash cooled in liquid nitrogen prior to data collection.

### Crystallographic Analysis of MSLN Variants

All X-ray diffraction datasets for MSLN-207, fl-MSLN/Fab(MORAb), and C-term/Fab(15B6) were collected at 100 K using beamline 22 (SER-CAT) or beamline 23 (GM/CA) at the Advanced Photon Source (APS), Argonne National Laboratory (ANL). Diffraction data images were processed using HKL2000 (36). The C-term/Fab(15B6) structure was solved by the molecular replacement (MR) method using the BALBES (37) program in the CCP4 program suite (38) and subsequently refined using Phenix (39) and REFMAC (40). The crystal structures of MSLN-207, fl-MSLN/Fab(MORAb), and fl-MSLN were also determined also by MR using MOLREP (41) and refined with Phenix and REFMAC. All structure models were built using the program COOT (RRID:SCR_014222; ref. 42).

### Glycan Analysis of Shed MSLN

The isolation of shed MSLN from either cultured A431/H9 cell culture media or patient ascites was carried out according to the procedure published previously (34). Briefly, an anti-MSLN column was prepared by covalently immobilizing 2 mg of SS1P, an anti-MSLN recombinant immunotoxin on a 1-mL column packed with N-hydroxy-succinimide–activated Sepharose resin. Shed MSLN derived from either A431/H9 cell culture supernatant or human ascites were then passed over the prepared anti-MSLN column. After washing the column with 10 column volumes of PBS, nonspecific binding proteins were removed by washing with sodium acetate buffer, pH 5.0, and MSLN was eluted with acetate buffer, pH 3.0 followed by immediate neutralization in 1 mol/L Tris-HCl, pH 8.0 buffer. Ascites samples used in this study were obtained from patients with mesothelioma seen at NIH, on an Institutional Review Board–approved protocol (ClinicalTrials.gov NCT 01950572). Written informed consent was obtained before patients were enrolled on the study. The study was conducted in accordance with the principles of the International Conference on Harmonization – Good Clinical Practice guidelines. Purified MSLN was analyzed using nano-LC/MS-MS. Briefly, affinity-purified samples were reduced, alkylated, digested with trypsin. The samples were injected to an Orbitrap Fusion Tribrid mass spectrometer through a nano-LC system (Thermo Fisher Scientific), and the glycopeptides were fragmentated by HCD-triggered CID program and further analyzed by manual annotation. For calculation of the relative abundances of glycans at each site, the AUC of each glycopeptide-derived mass was divided by the sum of all glycopeptides AUC.

### Data Availability

The atomic coordinates have been deposited in the Protein Data Bank (www.pdb.org) as follows: PDB ID code 7U9J for the MSLN-207, 7UED for the fl-MSLN/Fab of MORAb-009, 8CX3 for the fl-MSLN, and 7U8C for the C-term/Fab of Mab 15B6. The mass spectrometry proteomics data have been deposited to the ProteomeXchange Consortium via the PRIDE partner repository with the dataset identifier PXD034397.

## Results

### Identification of Suitable Fragments Leading to Structure Solution of fl-MSLN

We previously reported successful expression in insect cells and purification of the fully processed, fl-MSLN that contains 305 amino acid residues, spanning from residues E296 to T600 of the precursor protein (Fig. 1). Purified fl-MSLN is known to be glycosylated, as mutations removing the three predicted glycosylation sites significantly reduced the apparent molecular weight of the protein (24). Crystallization of the purified fl-MSLN yielded crystals that diffracted X-rays using synchrotron radiation, resulting in a dataset at a resolution of approximately 3.6 Å (Table 1). However, MR using a model predicted for MSLN (23) did not yield a correct solution. Extensive screening of heavy metal compounds also failed to produce a good quality derivative for phasing.

**TABLE 1:** Statistics on X-ray diffraction datasets and refined structural models

Dataset	MSLN-207	fl-MSLN	fl-MSLN/Fab(MORAb)	C-term/Fab(15B6)
Bound Mab	None	None	MORAb-009	15B6
*Data collection*				
Wavelength (Å)	0.97930	0.89197	1.0	1.03317
Space group	*C*2	*C2*	*I*422	*P*4_3_2_1_2
Cell dimension				
*a, b, c* (Å)	96.8, 78.6, 74.3	197.2, 279.3, 170.0	133.4, 133.4, 276.1	64.8, 64.8, 250.1
*α, β, γ* (°)	90, 108.1, 90	90, 125.1, 90	90, 90, 90	90, 90, 90
Resolution (Å)	50.0–2.09 (2.18–2.09)^a^	50–3.60 (3.73–3.60)	50.0–3.0 (3.11–3.00)	50.0–1.74 (1.80–1.74)
Total observations	65,861	196,634	66,407	1,471,831
Unique reflections	27,430	78,378	21,609	53,658
Redundancy	2.4	2.5	3.1	27.4
Completeness (%)	87.9 (70.4)	91.4 (78.1)	85.0 (70.1)	95.3 (70.4)
*R*_merge_^b^	0.103 (0.291)	0.123 (0.480)	0.119 (0.499)	0.092 (0.497)
I/σ_(I)_	5.6 (1.4)	4.9 (1.1)	6.4 (1.5)	35.0 (2.2)
Wilson B-factor (Å^2^)	14.2	99.8	60.6	29.7
*Refinement*				
Resolution (Å)	34.0–2.09 (2.17–2.09)	49.3–3.61 (3.70–3.61)	24.7–3.00 (3.14–3.00)	24.0–1.74 (1.77–1.74)
Reflections used in refinement	27,159	77,988	21,419	53,531
Working set	25,768 (1, 911)	74,040 (4, 264)	20,310 (2, 032)	50,761 (1, 661)
Test set	1,391 (99)	3,948 (227)	1,109 (102)	2,770 (88)
*R*_work_ (%)	17.1 (23.8)	26.8 (46.1)	20.1 (29.9)	20.4 (36.9)
*R*_free_ (%)	21.6 (29.0)	29.4 (47.2)	23.7 (29.0)	23.8 (37.2)
No. of copies in AU	2	6	1	1
No. of residues				
Heavy chain	0	0	220	214
Light chain	0	0	212	213
Mesothelin	418	1,485	287	15
Water	459	0	33	311
Saccharide residues	0	9	2	0
Sulfate/Phosphate	4	10	2	0
Glycerol	3	5	1	0
Average B-factor (Å^2^)	24.7	128.4	68.9	51.2
rms deviations				
Bond length (Å)	0.003	0.003	0.002	0.003
Bond angle (°)	0.604	0.797	0.553	0.534
Ramachandran plot				
Most favored	413 (99.8%)	1,385 (94.0%)	683 (95.8%)	422 (96.8%)
Allowed	1 (0.2%)	87 (5.9%)	28 (3.9%)	14 (3.2%)
Disallowed	0 (0.0%)	1 (0.1%)	2 (0.3%)	0 (0.0%)
*Deposition*	7U9J	8CX3	7UED	7U8C

^a^Numbers in the parentheses are statistics for outermost resolution shells.

^b^ *R*_merge_ is defined as Σ|*I*_h,i_ − <*I*_h_>|/Σ*I*_h,i_, where *I*_h,i_ is the intensity for *i*th observation of a reflection with Miller index h, and <*I*_h_> is the mean intensity for all measured *I*_h_s and Friedel pair.

Therefore, we adopted another approach to determine the structure of fl-MSLN, using MR method. This approach, however, required availability of structural models similar to that of MSLN (43). Because the only available structure for MSLN was its N-terminal antibody-binding fragment (MSLN-64; Fig. 1A; ref. 24), we decided to express and purify a series of MSLN fragments of different lengths so that each could serve as a template for larger fragment(s) using MR (Fig. 1A; Supplementary Table S2). We chose to express four fragments based on secondary structure predictions: MSLN-132 (296–426), MSLN-168 (296–462), MSLN-207 (296–501), and MSLN-263 (296–557), containing either two, three, or four cysteine residues (Fig. 1A; Supplementary Table S2). The N-terminal epitope was retained, allowing formation of a complex with the Fab fragment of Mab MORAb-009 for a larger molecular surface facilitating crystallization and providing a potentially better phasing model for the MR method.

**FIGURE 2:**
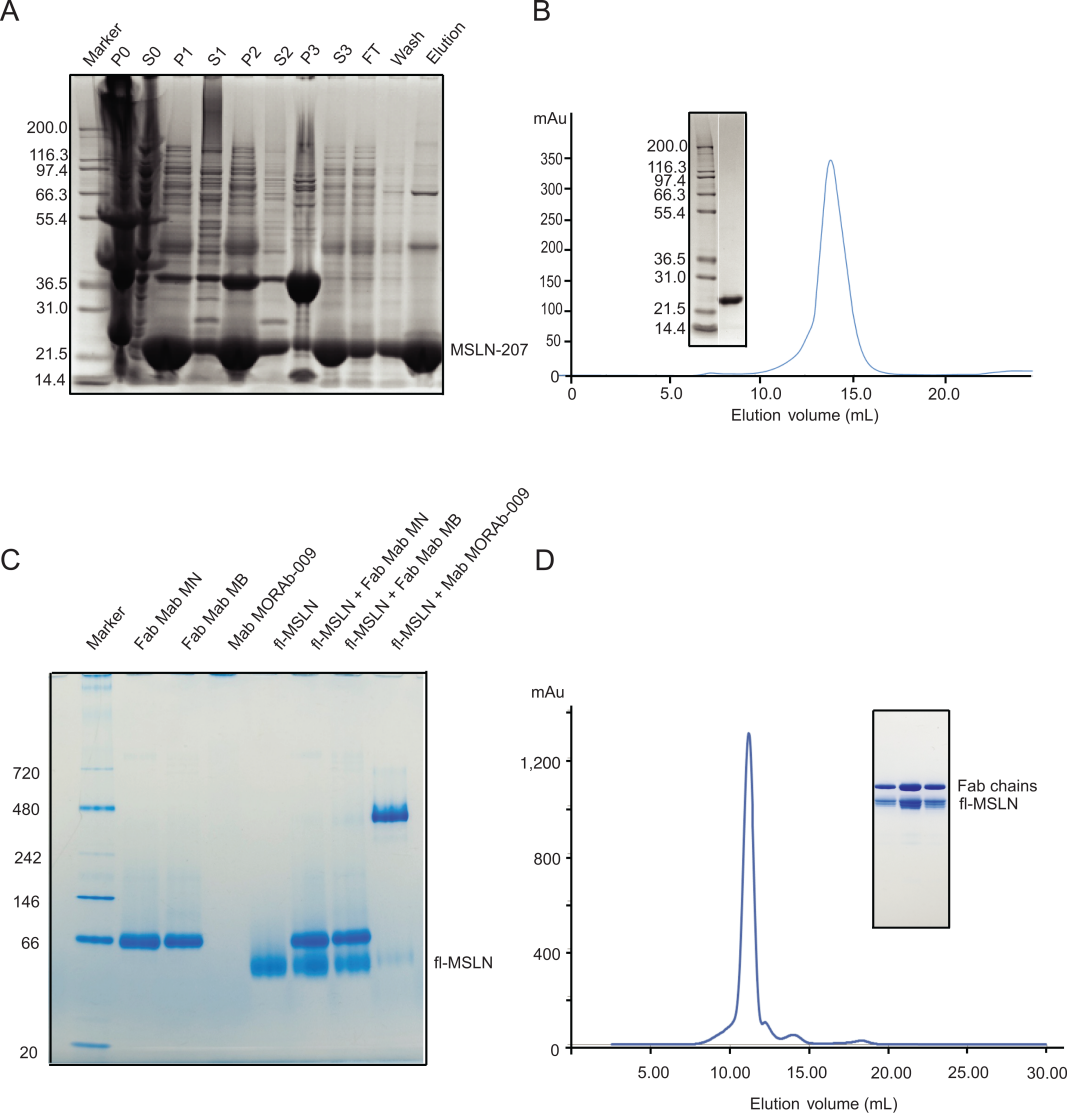
Purification of MSLN variants and interaction with various mAbs. **A,** Analysis of the purification process of MSLN-207 by SDS-PAGE. Cell lysate was centrifuged, and pellet and supernatant were labeled P0 and S0, respectively. The pellet was sonicated in wash buffer and pelleted down three times. Lanes labeled P1 and P2 are the pellets for the first two washes, and those labeled S1 and S2 are supernatants of the washes. Purified inclusion bodies were denatured by a solubilization buffer containing 8 mol/L urea followed by centrifugation, the pellet and supernatant are labeled P3 and S3, respectively. The supernatant was loaded on a Ni-NTA column with flow through (FT), washed (Wash), and eluted (Elution) components analyzed. **B,** Column-refolded MSLN-207 was further purified by SEC using a Superdex 200 column, which gave a peak at the position corresponding to 60 kDa globular proteins, consistent with the rod-shaped MSLN-207 that has a larger hydrodynamic radius. The inset shows the SDS-PAGE of the purified MSLN-207. **C,** BN-PAGE analysis of fl-MSLN interacting with various mAbs. Three mAbs were tested: Fab fragments of MN and MB (25) and mAb MORAb-009 (14) and their complexes with fl-MSLN. Mab MORAb-009 by itself appeared aggregated and could not be seen on the gel but could form a stable complex with fl-MSLN. Fab of either MN or MB did not form a complex with fl-MSLN. **D,** SEC profile of fl-MSLN in complex with Fab of MORAb-009. Purified Fab and fl-MSLN were mixed in a 1:1.1 ratio and incubated overnight at 4°C. The admixture was applied to a Superdex 200 column, and the inset shows the nonreducing SDS-PAGE of purified fl-MSLN/Fab(MORAb) complex, with the top band being Fab and the bottom band being fl-MSLN.

As shown in Supplementary Table S2, not all the MSLN fragments we designed led to successful structure determination. Like MSLN-64, the MSLN-132 was expressed in *E. coli* as a soluble protein and purified to homogeneity. Although both MSLN-132 and its complex with the Fab fragment of MORAb-009 yielded crystals, the diffraction qualities of these crystals were poor. Expressing longer fragments (MSLN-168, MSLN-207, and MSLN-263) in *E. coli* all produced inclusion bodies (Fig. 2). Refolding was successful only for the MSLN-207 construct (Fig. 2A), which includes all four conserved cysteine residues and seems to have the right size to maintain its structural integrity. Further purification by SEC yielded a single peak in the chromatogram, which rendered a single band by SDS-PAGE analysis (Fig. 2B).

### Structure of the MSLN-207 Fragment

**FIGURE 3:**
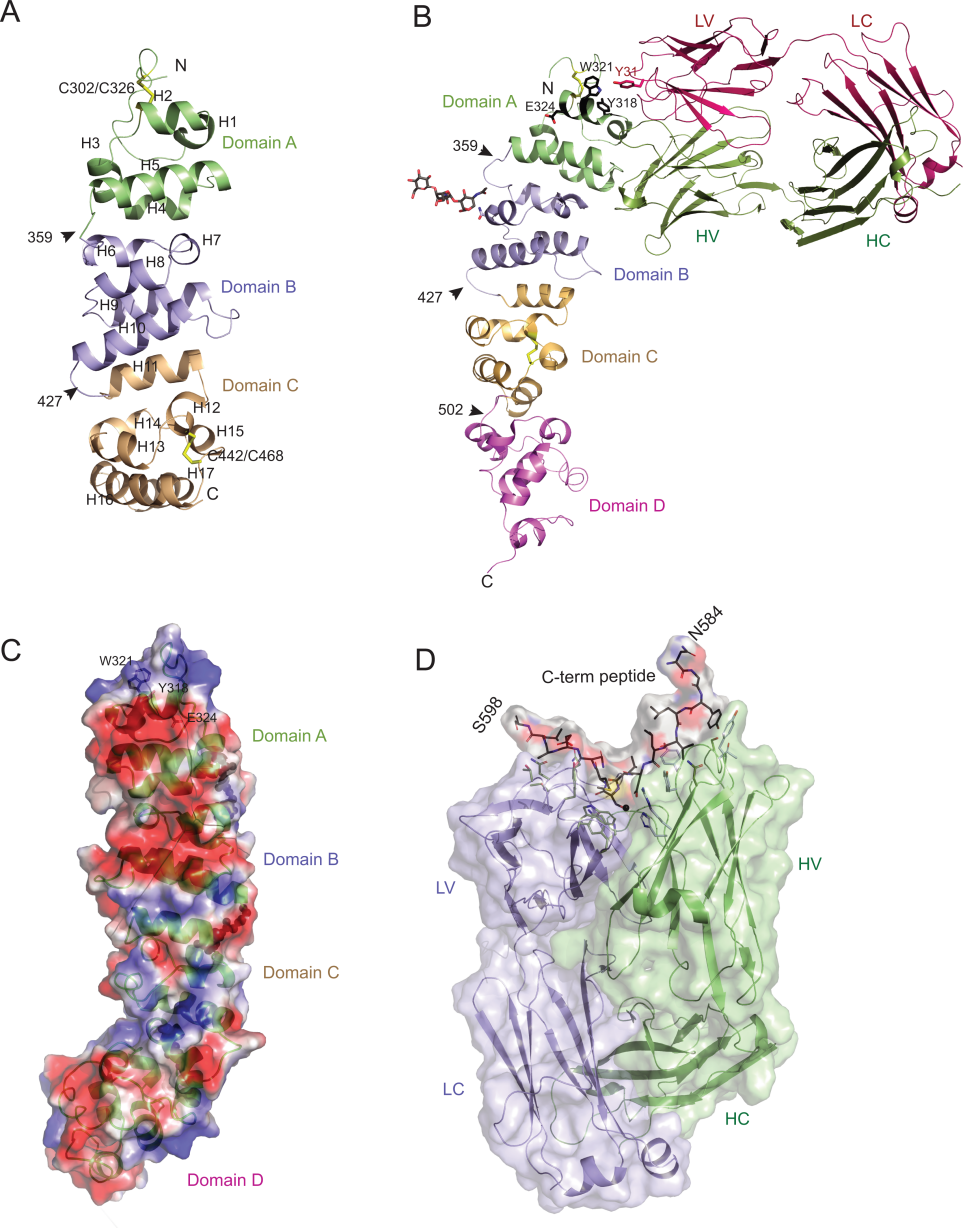
Structure of MSLN and its complex with Fab fragments. **A,** Ribbon diagram of the monomeric MSLN-207. The three more tightly associated subdomains are colored green (Domain A), purple (Domain B), and coral (Domain C), respectively. Also shown are the two disulfide bridges C302/C326 and C442/C468 rendered as stick models in yellow. The domain boundaries are indicated by black arrows and labeled. **B,** Ribbon presentation of the structure of fl-MSLN/Fab(MORAb) complex. Color codes for the subdomains A, B, and C are the same as in A. Subdomain D is colored magenta. The bound Fab fragment is shown as a cartoon with the L chain in red and H chain in green. The disaccharide bound to N388 is rendered as stick model with carbon atoms in black, nitrogen in blue, and oxygen in red. **C,** Electrostatic potential of fl-MSLN is mapped to its surface representation, with negative potential in red and positive potential in blue. This surface, half-transparent, is overlaid to the ribbon diagram of fl-MSLN. Corresponding subdomains are labeled. Residues known to interact with CA-125 are rendered as stick models and labeled. **D,** Structure of the C-term/Fab(15B6) complex is rendered as a cartoon overlaid with a transparent surface. This panel is reproduced from previously published data (34). The light chain (L) is colored purple, and the heavy chain (H) is colored green. The modeled C-terminal peptide from N584 to S598 is shown as a stick model with carbon atoms in black, nitrogen in blue, and oxygen in red. This stick model is superimposed with a surface model in gray.

The MSLN-207 fragment in the peak fraction crystallized in the monoclinic crystal form, and a diffraction dataset was obtained to 2.09 Å resolution (Table 1). MR search using the structural model of MSLN-64 was successful to find an initial solution, which was followed by iterative use of difference Fourier maps and manual modeling until a complete model of MSLN-207 was obtained (Fig. 3). There are two molecules of MSLN-207 in an asymmetric unit (AU). The two molecules (MSLN-207^A^ and MSLN-207^B^) are associated side by side in a head to tail manner, following noncrystallographic 2-fold symmetry (Supplementary Fig. S1A). This dimeric association is nonspecific, mediated by the C-terminal hexa-His-tag of MSLN-207^A^ to the N-terminal residues of the MSLN-207^B^. The two MSLN-207 molecules are superposable with a rms deviation for all CA atoms of 0.77 Å (Supplementary Table S3). Superpositions of MSLN-64 to either MSLN-207^A^ or MSLN-207^B^ gave an identical rms deviation of 0.45 Å (Supplementary Table S3). These structural alignments indicate that the N-terminal portion of the MSLN structure is rather rigid.

The structure of MSLN-207 consists of 17 short helices (Figs. 3A and 1B) that are arranged in a right-handed spiral, a superhelical topology that was predicted previously (23). Two disulfide bonds are formed for the cysteine pairs C302/C326 and C442/C468, and both pairs are conserved in our multiple sequence alignment (Fig. 1B). Interestingly, the structure of MSLN-207 appears to consist of three more tightly packed subdomains: Domains A (E296-L359), B (Y360-K427), and C (D428-G501; Fig. 3A). These subdomains define the boundaries corresponding to MSLN-64, MSLN-132, and MSLN-207, respectively, which could explain why the MSLN-168 fragment was not successfully refolded.

### Structure Determination of fl-MSLN in Complex with Fab of MORAb-009

Unlike MSLN-207, fl-MSLN was expressed in insect cells in a secreted form. Purified fl-MSLN was used to screen for a complex with mAb. Three mAbs were tested: MN, MB, and MORAb-009 (14, 24, 25). The Fab fragments of MN and MB did not appear to be suitable to form a complex with the purified recombinant fl-MSLN, which is consistent with the reported usage of these two mAbs for Western blot analysis, likely because of their interacting with unfolded MSLN (25). MORAb-009, on the other hand, was able to form a stable complex, as demonstrated by the BN-PAGE analysis (Fig. 2C), which was in agreement with the previously reported binding affinity (*K*_d_) of 5 nmol/L (24). The complex of fl-MSLN with Fab of MORAb-009 was purified to homogeneity and the peak fraction of fl-MSLN/Fab(MORAb) was monodispersed in solution, as judged by its SEC profile (Fig. 2D).

We were able to crystallize the fl-MSLN/Fab(MORAb) complex. Crystals of the complex diffracted X-rays using synchrotron radiation, leading to a dataset at 3.0 Å resolution (Table 1). The crystals possessed the symmetry of the space group *I*422 and featured one fl-MSLN/Fab(MORAb) complex per AU. The structure was determined by MR using the MSLN-64/Fab(MORAb) (PDB:4F3F) as a template for phasing (24). The final model was refined to *R*_work_ and *R*_free_ of 20.1% and 23.7%, respectively. In this crystal form, most of the crystal contacts were provided by the Fab fragment. Thus, the electron density for the fl-MSLN molecule is progressively less well defined in areas more distant from the bound antibody.

### The Structure of the fl-MSLN/Fab(MORAb) Complex

The fl-MSLN/Fab(MORAb) complex is L-shaped with the Fab bound to the side of the fl-MSLN spiral on Domain A (Fig. 3B). The model of fl-MSLN starts at residue K299 and ends with residue G585, missing N-terminal 3 and C-terminal 15 residues. As expected, the two disulfide bridges were preserved. Like the structure of MSLN-207, the fl-MSLN molecule also appears to be modular, adding a C-terminal subdomain termed Domain D (residues 502–585) that consists of six short helices (H18-H23; Fig. 1B). The C-terminus of MSLN beyond residue G585 is not visible in this structure. The addition of Domain D to the MSLN-207 fragment adds more curvature to the fl-MSLN molecule. Consequently, the superposition of MSLN-207 to its counterpart of the fl-MSLN gives rise to rms deviations of 1.52 and 1.28 Å, respectively, for MSLN-207^A^ and MSLN-207^B^ (Supplementary Table S3). Removing the Fab part of the model gives the fl-MSLN the shape of a letter J with the Domain D at an angle nearly 50° from the rest of the molecule (Fig. 3C). An electrostatic potential calculation indicates that the molecule has a largely negatively charged surface decorated with patches of positive charges. The N-terminal part centered on residue W321 where CA-125 binds is particularly positively charged (Fig. 3C).

**FIGURE 4:**
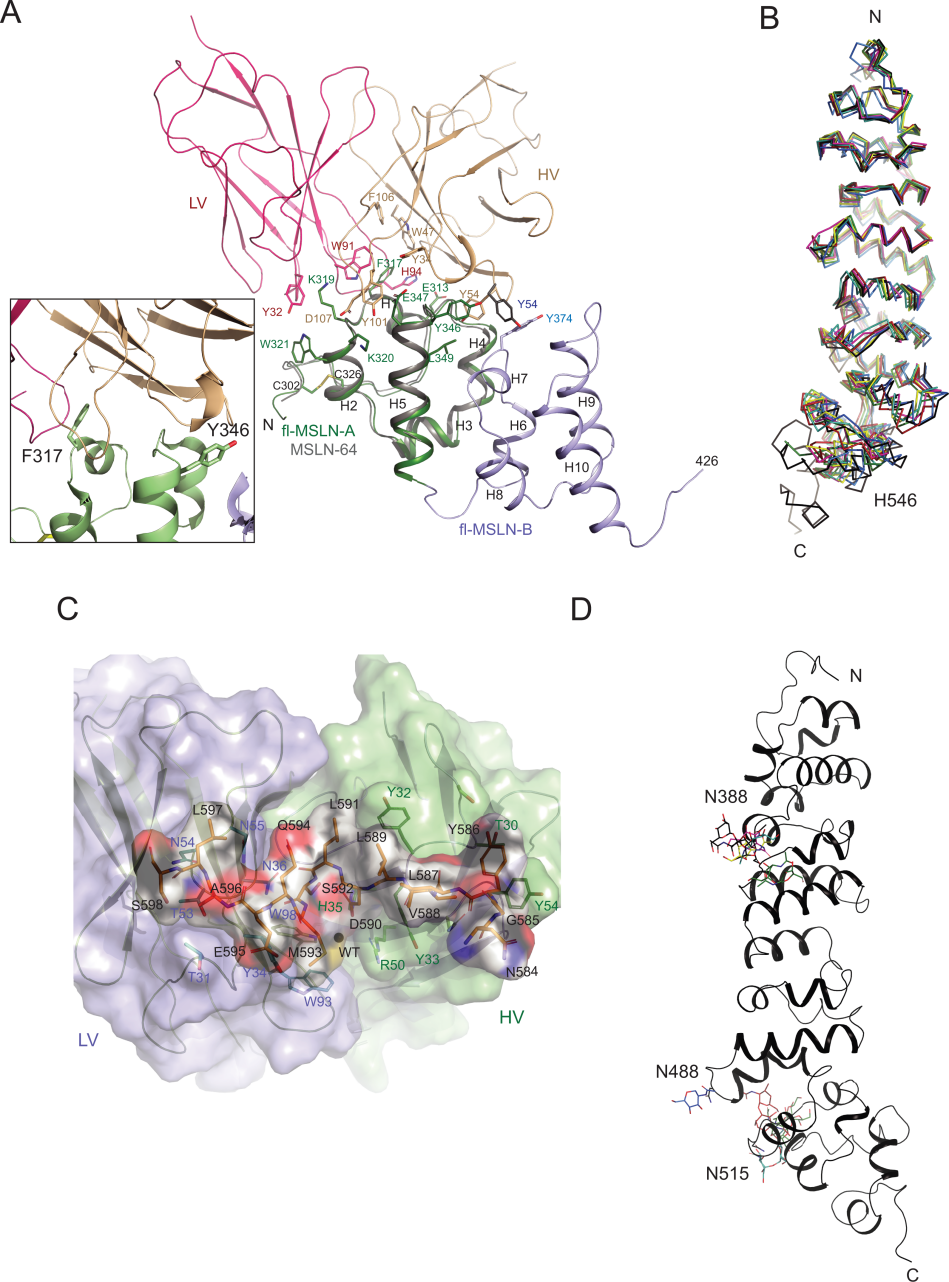
Flexibility of MSLN and its detailed interactions with therapeutic antibodies. **A,** Alignment of structures, based on Fab, between MSLN-64/Fab(MORAb) and fl-MSLN/Fab(MORAb) shows additional interactions of Fab with fl-MSLN. The structure of MSLN-64 in gray is shown as a cartoon model. The superposed fl-MSLN, also shown as cartoon, is colored in green for the Domain A (299–359) and in purple for the Domain B (359–426). The bound Fab fragment of MORAb-009 is rendered, in ribbon diagram, only for the variable domain with the light chain (LV) in red and the heavy chain (HV) in coral. Key residues providing interactions between MSLN-64 and Fab are given as stick models and labeled. Also highlighted as stick models are residues Y374 of fl-MSLN in Domain B and Y54 of heavy chain of the Fab. The latter undergoes conformational change in the fl-MSLN/Fab(MORAb) structure to avoid collision with the former. Inset: Mab MORAb-009 recognizes two nonconsecutive epitope determinants: one is centered on residue F317 and another on Y346. **B,** Alignment of structures of six fl-MSLN-245 and one fl-MSLN, all independently determined and rendered as Cα traces, shows large differences toward the C-terminal part of the structure. **C,** Magnified view of the interactions between the C-terminal peptide antigen of MSLN and the Fab of Mab 15B6, reproduced from previously published data (34). The structure of the peptide (N584-S598), shown as a Van der Waal's surface overlaid with its stick model with carbon in orange, nitrogen in blue, oxygen in red, and sulfur in yellow. The sequence of the peptide is labeled in black. The light (L) and heavy (H) chains of Fab (15B6) are shown as semitransparent molecular surfaces colored in purple and green, respectively, and overlaid with their cartoon diagrams. Residues of Fab (15B6) that interact with the peptide are shown as stick models and labeled. **D,** As in B, alignment of six structures of fl-MSLN-245 and one fl-MSLN led to superimposed sugar moieties attached to these protein molecules. Only the structure of the fl-MSLN is shown as a cartoon model. Modeled polysaccharides shown as stick models that are attached to the glycosylation sites at N388, N488, and N515.

The previously determined structure of MSLN-64/Fab(MORAb) showed that the complex is formed with MSLN-64 making contacts with both light and heavy chains of Fab (Fig. 4), though the major interactions are contributed by the heavy chain (24). The epitope recognized by MORAb-009, as revealed by the structure, consists of two nonconsecutive antigenic determinants (Fig. 4A; Supplementary Table S4A): the first is centered on helix H1 and extends on both sides from residue E313 (E18 in PDB:4F3F) of the N-terminal loop to the beginning of helix H2 (W321, annotated as W26 in PDB:4F3F). The second contains the loop between helices H4 and H5. The aromatic residue F317 penetrates deep into a hydrophobic pocket formed by residues from both light and heavy chains of the antibody and appears to provide essential hydrophobic and aromatic interactions (Fig. 4A). Peripheral interactions are mediated by mostly hydrophilic residues forming H-bonds and salt bridges (Supplementary Table S4A). As reported previously, the Mab MORAb-009 has a *K*_d_ value of 5 nmol/L toward fl-MSLN, which is one-fifth of that toward the MSLN-64 fragment (24) and suggests additional interactions between the antibody and the fl-MSLN. We compared the buried surface area (BSA) of MSLN-64/Fab(MORAb) with that of fl-MSLN/Fab(MORAb) (Supplementary Table S5) and found an increase of 12% from 1,714 Å^2^ to 1,918 Å^2^. This increase is largely due to additional interactions provided by the heavy chain with the Domain B of fl-MSLN because the BSA for the heavy chain of Fab is 616 Å^2^, greater than the 496 Å^2^ area for the MSLN-64/Fab(MORAb) complex (Supplementary Table S5), whereas BSA for the light chain remains basically unchanged.

Superposition of the structures of fl-MSLN/Fab(MORAb) and MSLN-64/Fab(MORAb) showed overall good alignment of the two but revealed several important adjustments made by residues in the Fab to accommodate additional interactions (Fig. 4A; Supplementary Table S4A). For example, the variable domain of heavy chain (HV domain) makes additional contact with helices H6 and H7 of Domain B in the fl-MSLN/Fab(MORAb) complex and the presence of Y374 clashes with the side chain of Y54 of HV domain in the conformation shown in MSLN-64/Fab(MORAb) complex, forcing it to adopt a new conformation (Fig. 4A).

### Determining the Structure of fl-MSLN

As mentioned above, a dataset was collected to about 3.6 Å resolution for the fl-MSLN. But the structure determination using this data was unsuccessful due to lack of both a suitable phasing model for MR and heavy metal derivatives. The structure solution of the fl-MSLN/Fab(MORAb) rekindled our effort to determine the structure of fl-MSLN. The crystal lattice of fl-MSLN was initially assigned to have the symmetry of the rhombohedral space group *R*32. MR with the fl-MSLN removed from the fl-MSLN/Fab(MORAb) complex structure as a search template revealed one molecule of MSLN in the crystallographic AU. However, subsequent structure refinement failed to converge, with severe collision with symmetry-related molecules toward the C-terminal part or Domain D of MSLN. The diffraction data set was reprocessed with the symmetry of space group *C*2 (Table 1). An MR search found six MSLN molecules per AU and refinement proceeded successfully only when the dataset was corrected for twining. The resulting electron densities were sufficiently clear to allow modeling of at least 245 residues of MSLN from K299 to L543 for all six molecules, missing about 50 C-terminal residues due to disorder (Supplementary Fig. S1B–E). The six truncated fl-MSLN models, here after referred to as fl-MSLN-245, in the AU were organized as a triangle, with each side formed by a pair of fl-MSLN-245 molecules arranged tail to tail (Supplementary Fig. S1B and S1C). Two of the fl-MSLN-245 triangles related by a 2-fold symmetry axis formed a double stack (Supplementary Fig. S1D and S1E) that was the basic motif for crystal formation.

Superpositions of the six independently determined fl-MSLN-245 models to the fl-MSLN gave rise to an average rms deviation of 1.3 Å, whereas limiting the superposition to residues from K299 to K427, that is, from Domains A to C, reduced rms deviation significantly to about 0.55 Å (Fig. 4B; Supplementary Table S3). Consistent with the observation concerning the fl-MSLN, different MSLN-245 models display bending of the subdomain D to different degrees. The lack of a quality electron density for the last 50 C-terminal residues of MSLN beyond residue H546 suggests the C-terminal portion of MSLN is conformationally flexible. In contrast, fl-MSLN with bound Fab(MORAb) gives a more complete MSLN structure of 287 residues (K299-G585) from a total of 305 residues in the construct. Whether the binding of Fab at the N-terminal domain of MSLN helps to stabilize the C-terminal part of MSLN remains to be seen.

### MSLN Consists of Mixed ARM and HEAT Repeats

**FIGURE 5:**
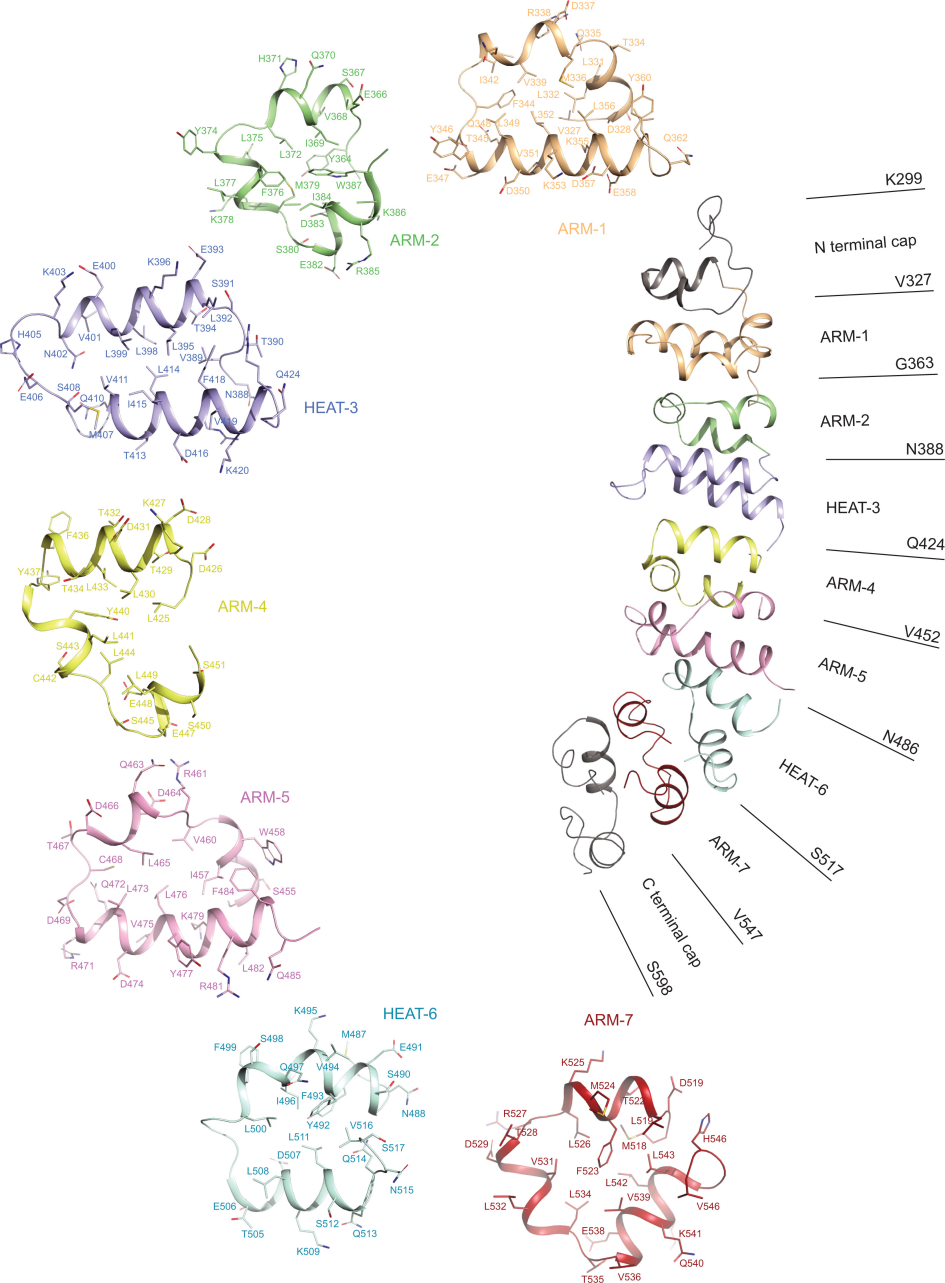
ARM/HEAT repeats in fl-MSLN. The structure of fl-MSLN is rendered as cartoon with each ARM/HEAT motif given a unique color and label. The boundary of each motif is also given. Each repeat is also rendered individually as cartoon with amino acid residues shown as stick models and labeled.

MSLN and its precursor were predicted to have an ARM-repeat solenoid (23). ARM-repeat structures are made of tandem repeats of approximately 30–40 residue-long helix-turn-helix motifs. A canonical ARM repeat consists of three helices, denoted H1, H2, and H3 to make a full circle. The H2 and H3 helices pack against each other in an antiparallel fashion and are roughly perpendicular to the shorter H1 helix, with a sharp bend between helices H1 and H2 mediated by a conserved glycine residue (44). In ARM-repeat proteins, neighboring repeats stack together into a single domain with a continuous hydrophobic core, forming an elongated superhelix. We checked these properties of ARM repeats against our MSLN structure by subdividing it into repeating units, each forming a complete circle. For MSLN, there are seven repeating units in addition to the N- and C-terminal caps (Fig. 5). Among them five contain three helices each and qualify for the ARM repeats. They are named ARM-1, 2, 4, 5, and 7. Two repeating units contain only two helices each, which are classified as HEAT motif and named as HEAT-3 and 4. Thus, MSLN is neither an ARM nor a HEAT structure but rather a structure containing a mix of both motifs.

These ARM and HEAT repeats of MSLN have different lengths ranging from 27 to 37 residues. Sequence and structural alignment between pairs of these repeats showed little detectable homologies. Common to all repeats is the concentration of hydrophobic residues such as Leu, Ile, Val, and Phe forming hydrophobic cores (Fig. 5). Hydrophilic and charged residues are distributed at the periphery of each unit. We were curious of why some of our constructs such as MSLN-64, MSLN-132, and MSLN-207 could be purified, while others like MSLN-168 and MSLN-263 could not. Examining hydrophobic interactions between neighboring pairs of repeats (Supplementary Table S4B) revealed no hydrophobic interactions between ARM-1 and ARM-2 and between ARM-5 and HEAT-6, which explained why MSLN-64 and MSLN-207 could be successfully purified and crystallized. All other pairs have hydrophobic interactions, albeit to different degrees. For example, MSLN-168 terminates at the interface between ARM-4 and ARM-5, which disrupts the disulphide bond between C442 and C468 and exposed a large patch of hydrophobic area. Similarly exposed hydrophobic area can be found for the construct MSLN-263. The construct MSLN-132 was purified and crystallized. But crystals of MSLN-132 diffracted poorly (Supplementary Table S2). Interestingly, MSLN-132 terminates at the interface between HEAT-3 and ARM-4, where H2 and H3 of ARM-4 have no contact with HEAT-3 and large part of the HEAT-3 surface was exposed to solvent even in the fl-MSLN.

### Structure of the C-terminal Shedding-resistant Peptide Complexed with Fab of mAb 15B6

The investigation of the mechanism of MSLN shedding from the cell surface led to identification of a C-terminal region of MSLN (residue 582–598; Fig. 1B) that contains majority of the major protease cleavage sites and the development of mAb 15B6 targeting this region (45). mAb 15B6 is a mouse isotype IgG2bλ, which was found to prevent MSLN shedding. Our fl-MSLN structure only covers the first two residues of this 17-amino acid peptide (Fig. 1B), which prompted us to obtain a structure of the peptide in complex with Mab 15B6. A synthetic 17-residue peptide (residue 582–598; Fig. 1B) was mixed with Fab of mAb 15B6 to form the peptide/Fab complex, named C-term/Fab(15B6), which was successfully crystallized. An X-ray diffraction dataset was obtained to 1.74 Å resolution and was used to obtain the structure of the complex, again by MR, which was subsequently refined to *R*_work_ and *R*_free_ of 20.4% and 23.8%, respectively (Table 1).

The experimental electron density was fit first with a Fab structure model (PDB:3RIF), followed by manual model adjustments based on the sequence of 15B6. The final model of the Fab(15B6) contains a 213-residue light chain and a 214-residue heavy chain (Figs. 1C and 3D). Upon completion of Fab(15B6) model building, there is a piece of unmodeled density spanning across the complementarity-determining regions (CDR) of the Fab(15B6), and this coincides with the positive difference density map (*F*_o_-*F*_c_), into which a model of the 15-residue peptide beginning with N584 and ending with S598 of the MSLN sequence could be placed (Fig. 1B). The bound peptide adopts an extended conformation with a linear epitope consisting of 10 residues (from Y586 to L597) of the 15-residue model (Fig. 3D). The two hydrophobic residues L587 and L589 within the epitope have their side chains pointing away from the Fab, thus not contributing to the binding interface (Fig. 4C). The contacts between the bound peptide and Fab are dominated by H-bonding and other hydrophilic interactions (Supplementary Table S4C).

### Glycosylation Sites and Glycan Structure Analysis of MSLN

Isolated full-length recombinant MSLN from insect cell expression system is glycosylated. The elucidation of MSLN structure is not complete without its glycan structures. In the context of developing more effective anti-MSLN antibodies, understanding the glycan structures associated with MSLN is necessary, as the binding of CA-125 to MSLN, which facilitates metastasis of ovarian tumors, appears to be dependent on glycosylation states of MSLN (16). Furthermore, CAR-T cells generated on the basis of the N-terminal region (296–390) or the glycosylated C-terminal region (487–598) demonstrated differential killing of tumor cells (22). We thought it might be informative to study the glycosylation pattern of MSLN, although no clear roles have been identified for MSLN N-glycans so far.

Human MSLN contains three predicted N-glycosylation sites: N388, N488, and N515. Among them, N388 and N515 are conserved in a sequence alignment with mouse and rat genes (Fig. 1B). Because the fl-MSLN used in our structural studies was expressed in insect cells and was shown to be glycosylated (24), we took the opportunity to examine oligosaccharide attachments after the fl-MSLN structures were obtained. All three predicted glycosylation sites were observed to have attached glycans (Supplementary Table S6; Fig. 4D), although no more than one site in each molecule was found glycosylated in the crystals (Supplementary Table S6). These attached sugar moieties are represented by strong difference densities sufficiently large to accommodate at least one N-acetyl-D-glucosamine residue (GlcNAc) and in some cases up to three saccharide residues including two GlcNAc and one β-D-mannose (BMA) residues that can be included in the model. Thus, fl-MSLN purified from insect cells has rather uniform non-sialic, paucimannosic glycan side chains (Supplementary Table S6), which agrees with the glycan analyses of recombinant proteins expressed from sf9 and Hi5 cells reported in the literature (46). Because of the limited resolution, it is not possible to differentiate different linkages between sugar moieties, such as 1,4-acetylglucosaminylation, 1,6- or 1,3-fucosylation. The asparagine residues N388 is the site most frequently found glycosylated. Two chains were glycosylated at N515. Only one chain was found with a glycosylated N488. Superposition of seven independently determined fl-MSLN structures [one from the fl-MSLN/Fab(MORAb) crystal and six from fl-MSLN crystal] shows different conformations of the sugar moieties even at the same site (Fig. 4D), suggesting that crystal contacts are a major factor contributing to the conformations of attached oligosaccharides.

**FIGURE 6:**
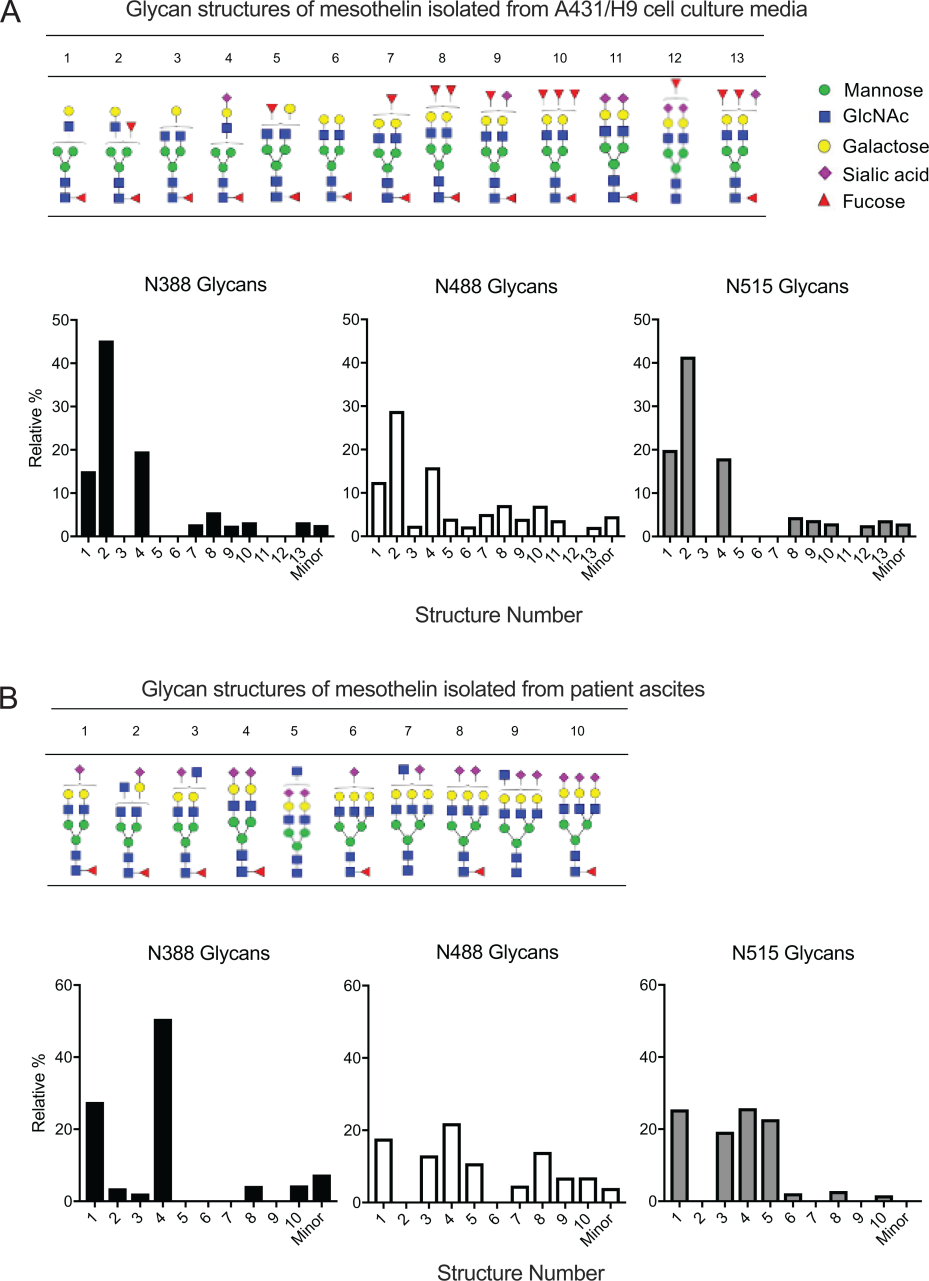
N-glycan signatures present in MSLN isolated from A431/H9 cell culture supernatant and from patient ascites. Affinity purified MSLN was reduced, alkylated, desalted, and digested with trypsin for nano-LC/MS-MS analysis. Monosaccharide symbols used as follows: triangle, fucose; blue square, N-acetylglucosamine; green circle, mannose; yellow circle, galactose; purple diamond, N-acetylneuraminic acid. **A,** MSLN shed from A431/H9 cells. Top: N-glycan structures identified along the MSLN peptide backbone. Bottom: Site-specific percentages of glycoforms present at each N-glycosylation residue: N388 (left), N488 (middle), and N515 (right). **B,** MSLN isolated from patient ascites. Top: N-glycan structures identified along the MSLN peptide backbone. Bottom: Site-specific percentages of glycoforms present at each N-glycosylation residue: N388 (left), N488 (middle), and N515 (right).

Considering our fl-MSLN was produced in insect cells, whose glycobiological pathway is simpler than that of mammalian cells (46), we wanted to confirm if the glycosylation pattern of MSLN of human origin would be the same. We carried out quantitative site-specific N-linked glycan characterization of MSLN shed from A431/H9 cells and from an ascites sample from a human patient by a glycomic and glycoproteomic approach. A431/H9 is a human MSLN-transfected A431 cell line (epidermoid cancer) that highly expresses and sheds MSLN (34). Reduced and alkylated MSLN was trypsin digested, followed by nano-LC/MS-MS analysis. Consistent with the crystallographic analysis, we identified substantial glycan occupancy at all three predicted N-glycosylation sites for MSLN from both A431/H9 cells and patient ascites (Fig. 6). Glycosylation of MSLN from A431/H9 cells is of complex type with N-Glycan fucosylation at all sites, along with minor sialylated antennary N-glycans (Fig. 6A). Glycosylation of MSLN from patient ascites is also of complex type but heavily sialylated and moderately fucosylated (Fig. 6B). These features are distinct from MSLN expressed from insect cells.

## Discussion

**FIGURE 7:**
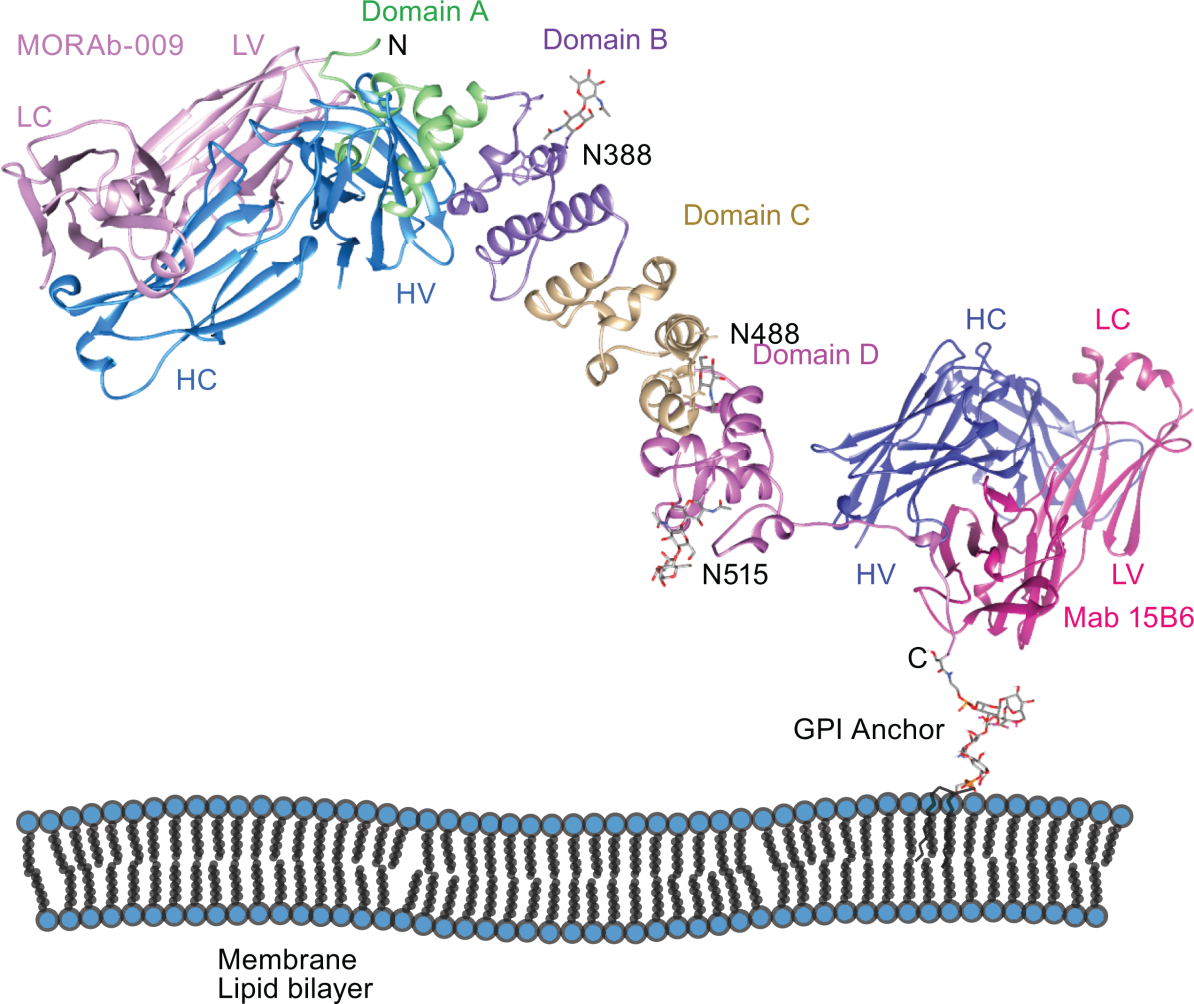
Structure of MSLN and its interactions with antibodies. A model of MSLN constructed from the structures of fl-MSLN/Fab(MORAb) and C-term/Fab(15B6), supplemented with attached saccharides from the structures of fl-MSLN and MSLN-245. In addition, the C-terminus of MSLN is modeled with a GPI-linkage that allows the protein to be anchored to the membrane bilayer. The MSLN model is rendered as a ribbon diagram with the four subdomains colored differently and labeled. The Fab fragment of MORAb-009 is shown as a ribbon diagram and the light and heavy chains are colored pink and blue, respectively. The Fab of the C-terminal-specific Mab 15B6 is also rendered as ribbon with the light chain in red and heavy chain in purple. Polysaccharides attached to the glycosylation sites are shown as stick models with carbon atoms in gray, nitrogen in blue, and oxygen in red.

In this work, we expressed and successfully purified a MSLN fragment (MSLN-207, E296-G501) and fl-MSLN (E296-T600). Our structure determination effort yielded the structural models of MSLN-207 (K299-G501), the fl-MSLN-245 (K299-H546), and the fl-MSLN (K299-G585)/Fab (Mab MORAb-009), missing the C-terminal 15 residues in the full-length construct. We also determined the structure of the C-terminal peptide (N584-S598) in complex with Fab of Mab 15B6. These structures allowed us to make a full description of the MSLN structure from K299 to S598 (Fig. 7). From structural superposition, we observed that the N-terminal part of MSLN (K299-N486; Fig. 4B; Supplementary Table S3) is relatively rigid, thanks to the two pairs of disulfide bridges. The C-terminal part of MSLN (M487-S598) is more flexible, as evidenced by the disordered ARM/HEAT repeat 7 and C-terminal cap in the fl-MSLN structure (Fig. 4B). It is conceivable that MSLN anchored on the cell surface by GPI is also highly flexible. However, we observed that the C-terminal part of MSLN can be stabilized when its N-terminal domain is complexed with Fab of MORAb-009, although we cannot determine whether this stabilization effect is indeed derived from Fab binding or due to crystal contacts.

The structure elucidation of MSLN was previously attempted by computational modeling approaches. We compared our fl-MSLN structure with an early version of a predicted structure (23) and with a more recent prediction by AlphaFold2 (ref. 47; Supplementary Fig. S2A and S2B). Both models correctly predicted the solenoidal structure of MSLN. Clearly, significant progress has been made in predicting protein structures over the years, as the model by AlphaFold2 (Supplementary Fig. S2B) is more accurate than that of the earlier model (Supplementary Fig. S2A), when compared with the experimental model, in terms of rms deviations (3.0 vs. 15.8 Å over the MSLN residue range of 300–585) and boundaries of predicted secondary structures. However, the computational approach appears insufficient to predict interactions with bound ligand, which is exemplified by the prediction of the structure of the C-terminal peptide/15B6 complex (Supplementary Fig. S2C).

ARM/HEAT proteins should not be considered as being composed of a series of independent folding units aligned like beads on a string, because they have a single rodlike domain with a continuous hydrophobic core formed by interactions between adjacent ARM/HEAT repeats. Well-known ARM/HEAT repeat proteins are importin-α, also known as karyopherin, which recognizes nuclear localization signals (48) and the cell adhesion molecule β-catenin (49). Although ARM/HEAT-repeat proteins are involved in various cellular processes, one common function is that of mediating important protein–protein interactions. Structures of ARM/HEAT type proteins determined experimentally contain 10 to 18 repeats. A Dali search based on the fl-MSLN structure produced a list of similar structures in the protein structure database (Supplementary Table S7; ref. 50). Except for the first hit, the structure of MSLN-64 reported previously (24), all other top-ranked structures have ARM/HEAT repeats but share very low sequence identity with MSLN. Most of these ARM/HEAT proteins form complexes with other proteins.

On the basis of the definition of the ARM/HEAT repeat, the fl-MSLN contains seven ARM/HEAT repeats sandwiched between the N- and C-terminal caps (Fig. 5). Each repeat consists of either two or three helices. Like other ARM/HEAT proteins, the solenoid of MSLN is curved, containing a concave and a convex surface (Fig. 5). All glycosylation sites are located on the convex side. The Mab MORAb-009 binding site is located on the concaved surface partially overlapping with the CA-125 binding site (Fig. 7). The previous characterization of CA-25 binding to MSLN identified essential residues located on the N-terminal part of MSLN (18), which can be mapped to the MSLN structure. The CA-125 binding surface centered on residue W321 (Fig. 3C). The interaction between CA-125 and MSLN was shown to be specific, as other mucin molecules do not interact with MSLN (16, 17). The chemical nature of their interactions remains to be elucidated. It remains to be seen how the Thy1 protein, a highly glycosylated cell surface protein identified to form a complex with MSLN in activated portal fibroblast, interacts with MSLN (20). Moreover, it is conceivable that MSLN may have other binding partners not yet identified.

Targeting MSLN for immunotherapy is based on observations of increased MSLN-specific antibodies detected in sera of about 40% of patients with mesothelioma and 42% of patients with ovarian cancer, indicating that antibody responses to MSLN was correlated with higher expression levels of MSLN on the surface of cancer cells (12). Promising clinical results from targeting MSLN in patients with cancer have stimulated searches for more effective mAbs, which can benefit from our structural studies of MSLN. Superposition of a total of seven independently determined MSLN molecules from this study (Fig. 4B) demonstrates that although overall MSLN is a flexible molecule, it has several built-in rigid blocks or subdomains (Domains A, B, and C; Figs. 3A, 3B, 5; Supplementary Fig. S3) that can potentially be used as unique antigenic epitopes for antibody targeting. Indeed, most high-affinity mAbs produced targeted the N-terminal region of MSLN in Domains A and B (Fig. 7; Supplementary Fig. S3); very few were found binding to Domains C and D (51).

Interestingly, elevated serum MSLN was found in most patients with mesothelioma (71%) and ovarian cancer (67%; ref. 33). Blood MPF and MSLN levels were correlated, with modest accuracy, for malignant pleural mesothelioma and lung cancer (52). The increased serum MSLN results from shedding of MSLN from the cell surface due to the activity of membrane bound extracellular proteases (32). Our structure confirmed that these proteolytic sites in the C-terminal cap region of the MSLN could be targeted by monoclonal antibodies to shield these sites from protease attacks.

## Supplementary Material

Table S1-S7 Table S1: Plasmids and primers used for protein expression.
Table S2: Characterization of MSLN expression constructs.
Table S3: Structural alignments of MSLN models.
Table S4: Lists of interactions.
Table S5: Buried surface area for three MSLN-Fab complexes.
Table S6: Observed saccharide attachments at predicted glycosylation sites.
Table S7: Dali search result.

Figure S1 Arrangement of MSLN molecules in the crystal.

Figure S2 Comparison of experimental MSLN structure with those of computational models.

Figure S3 Mapping epitopes of monoclonal antibodies to the structure of MSLN.

## References

[1] Chang K , Pastan I, Willingham MC. Isolation and characterization of a monoclonal antibody, K1, reactive with ovarian cancers and normal mesothelium. Int J Cancer 1992;50:373–81. 1735605 10.1002/ijc.2910500308

[2] Chang K , Pastan I. Molecular cloning of mesothelin, a differentiation antigen present on mesothelium, mesotheliomas, and ovarian cancers. Proc Natl Acad Sci U S A 1996;93:136–40. 8552591 10.1073/pnas.93.1.136 PMC40193

[3] Argani P , Iacobuzio-Donahue C, Ryu B, Rosty C, Goggins M, Wilentz RE, . Mesothelin is overexpressed in the vast majority of ductal adenocarcinomas of the pancreas: identification of a new pancreatic cancer marker by serial analysis of gene expression (SAGE). Clin Cancer Res 2001;7:3862–8. 11751476

[4] Ordóñez NG . Value of mesothelin immunostaining in the diagnosis of mesothelioma. Mod Pathol 2003;16:192–7. 12640097 10.1097/01.MP.0000056981.16578.C3

[5] Hassan R , Bera T, Pastan I. Mesothelin: a new target for immunotherapy. Clin Cancer Res 2004;10:3937–42. 15217923 10.1158/1078-0432.CCR-03-0801

[6] Ho M , Bera TK, Willingham MC, Onda M, Hassan R, FitzGerald D, . Mesothelin expression in human lung cancer. Clin Cancer Res 2007;13:1571–5. 17332303 10.1158/1078-0432.CCR-06-2161

[7] Hassan R , Kreitman RJ, Pastan I, Willingham MC. Localization of mesothelin in epithelial ovarian cancer. Appl Immunohistochem Mol Morphol 2005;13:243–7. 16082249 10.1097/01.pai.00000141545.36485.d6

[8] Hassan R , Laszik ZG, Lerner M, Raffeld M, Postier R, Brackett D. Mesothelin is overexpressed in pancreaticobiliary adenocarcinomas but not in normal pancreas and chronic pancreatitis. Am J Clin Pathol 2005;124:838–45. 16416732

[9] Hassan R , Thomas A, Alewine C, Le DT, Jaffee EM, Pastan I. Mesothelin immunotherapy for cancer: ready for prime time? J Clin Oncol 2016;34:4171–9. 27863199 10.1200/JCO.2016.68.3672 PMC5477819

[10] Hassan R , Miller AC, Sharon E, Thomas A, Reynolds JC, Ling A, . Major cancer regressions in mesothelioma after treatment with an anti-mesothelin immunotoxin and immune suppression. Sci Transl Med 2013;5:208ra147. 10.1126/scitranslmed.3006941 PMC6369691 24154601

[11] Yeo D , Castelletti L, van Zandwijk N, Rasko JEJ. Hitting the bull's-eye: mesothelin's role as a biomarker and therapeutic target for malignant pleural mesothelioma. Cancers 2021;13:332. 34439085 10.3390/cancers13163932 PMC8391149

[12] Ho M , Hassan R, Zhang J, Wang QC, Onda M, Bera T, . Humoral immune response to mesothelin in mesothelioma and ovarian cancer patients. Clin Cancer Res 2005;11:3814–20. 15897581 10.1158/1078-0432.CCR-04-2304

[13] Yen MJ , Hsu CY, Mao TL, Wu TC, Roden R, Wang TL, . Diffuse mesothelin expression correlates with prolonged patient survival in ovarian serous carcinoma. Clin Cancer Res 2006;12:827–31. 16467095 10.1158/1078-0432.CCR-05-1397

[14] Chowdhury PS , Pastan I. Improving antibody affinity by mimicking somatic hypermutation *in vitro*. Nat Biotechnol 1999;17:568–72. 10385321 10.1038/9872

[15] Scholler N , Fu N, Yang Y, Ye Z, Goodman GE, Hellstrom KE, . Soluble member(s) of the mesothelin/megakaryocyte potentiating factor family are detectable in sera from patients with ovarian carcinoma. Proc Natl Acad Sci U S A 1999;96:11531–6. 10500211 10.1073/pnas.96.20.11531 PMC18068

[16] Rump A , Morikawa Y, Tanaka M, Minami S, Umesaki N, Takeuchi M, . Binding of ovarian cancer antigen CA125/MUC16 to mesothelin mediates cell adhesion. J Biol Chem 2004;279:9190–8. 14676194 10.1074/jbc.M312372200

[17] Gubbels JA , Belisle J, Onda M, Rancourt C, Migneault M, Ho M, . Mesothelin-MUC16 binding is a high affinity, N-glycan dependent interaction that facilitates peritoneal metastasis of ovarian tumors. Mol Cancer 2006;5:50. 17067392 10.1186/1476-4598-5-50 PMC1635730

[18] Kaneko O , Gong L, Zhang J, Hansen JK, Hassan R, Lee B, . A binding domain on mesothelin for CA125/MUC16. J Biol Chem 2009;284:3739–49. 19075018 10.1074/jbc.M806776200 PMC2635045

[19] Avula LR , Rudloff M, El-Behaedi S, Arons D, Albalawy R, Chen X, . Mesothelin enhances tumor vascularity in newly forming pancreatic peritoneal metastases. Mol Cancer Res 2020;18:229–39. 31676721 10.1158/1541-7786.MCR-19-0688 PMC8139242

[20] Koyama Y , Wang P, Liang S, Iwaisako K, Liu X, Xu J, . Mesothelin/mucin 16 signaling in activated portal fibroblasts regulates cholestatic liver fibrosis. J Clin Invest 2017;127:1254–70. 28287406 10.1172/JCI88845 PMC5373891

[21] Kojima T , Oh-eda M, Hattori K, Taniguchi Y, Tamura M, Ochi N, . Molecular cloning and expression of megakaryocyte potentiating factor cDNA. J Biol Chem 1995;270:21984–90. 7665620 10.1074/jbc.270.37.21984

[22] Zhang Z , Jiang D, Yang H, He Z, Liu X, Qin W, . Modified CAR T cells targeting membrane-proximal epitope of mesothelin enhances the antitumor function against large solid tumor. Cell Death Dis 2019;10:476. 31209210 10.1038/s41419-019-1711-1 PMC6572851

[23] Sathyanarayana BK , Hahn Y, Patankar MS, Pastan I, Lee B. Mesothelin, stereocilin, and otoancorin are predicted to have superhelical structures with ARM-type repeats. BMC Struct Biol 2009;9:1. 19128473 10.1186/1472-6807-9-1 PMC2628672

[24] Ma J , Tang WK, Esser L, Pastan I, Xia D. Recognition of mesothelin by the therapeutic antibody MORAb-009: structural and mechanistic insights. J Biol Chem 2012;287:33123–31. 22787150 10.1074/jbc.M112.381756 PMC3460419

[25] Onda M , Willingham M, Nagata S, Bera TK, Beers R, Ho M, . New monoclonal antibodies to mesothelin useful for immunohistochemistry, fluorescence-activated cell sorting, Western blotting, and ELISA. Clin Cancer Res 2005;11:5840–6. 16115924 10.1158/1078-0432.CCR-05-0578

[26] Hassan R , Ebel W, Routhier EL, Patel R, Kline JB, Zhang J, . Preclinical evaluation of MORAb-009, a chimeric antibody targeting tumor-associated mesothelin. Cancer Immun 2007;7:20. 18088084 PMC2935758

[27] Zhang Y , Xiang L, Hassan R, Paik CH, Carrasquillo JA, Jang BS, . Synergistic antitumor activity of taxol and immunotoxin SS1P in tumor-bearing mice. Clin Cancer Res 2006;12:4695–701. 16899620 10.1158/1078-0432.CCR-06-0346

[28] Hassan R , Schweizer C, Lu KF, Schuler B, Remaley AT, Weil SC, . Inhibition of mesothelin-CA-125 interaction in patients with mesothelioma by the anti-mesothelin monoclonal antibody MORAb-009: implications for cancer therapy. Lung Cancer 2010;68:455–9. 19744744 10.1016/j.lungcan.2009.07.016 PMC2864325

[29] Haas AR , Tanyi JL, O'Hara MH, Gladney WL, Lacey SF, Torigian DA, . Phase I study of lentiviral-transduced chimeric antigen receptor-modified T cells recognizing mesothelin in advanced solid cancers. Mol Ther 2019;27:1919–29. 31420241 10.1016/j.ymthe.2019.07.015 PMC6838875

[30] Pastan I , Hassan R, Fitzgerald DJ, Kreitman RJ. Immunotoxin therapy of cancer. Nat Rev Cancer 2006;6:559–65. 16794638 10.1038/nrc1891

[31] Zhang Y , Chertov O, Zhang J, Hassan R, Pastan I. Cytotoxic activity of immunotoxin SS1P is modulated by TACE-dependent mesothelin shedding. Cancer Res 2011;71:5915–22. 21775520 10.1158/0008-5472.CAN-11-0466 PMC3165076

[32] Liu X , Chan A, Tai CH, Andresson T, Pastan I. Multiple proteases are involved in mesothelin shedding by cancer cells. Commun Biol 2020;3:728. 33262421 10.1038/s42003-020-01464-5 PMC7708464

[33] Hassan R , Remaley AT, Sampson ML, Zhang J, Cox DD, Pingpank J, . Detection and quantitation of serum mesothelin, a tumor marker for patients with mesothelioma and ovarian cancer. Clin Cancer Res 2006;12:447–53. 16428485 10.1158/1078-0432.CCR-05-1477

[34] Liu X , Onda M, Watson N, Hassan R, Ho M, Bera TK, . Highly active CAR T cells that bind to a juxtamembrane region of mesothelin and are not blocked by shed mesothelin. Proc Natl Acad Sci U S A 2022;119:e2202439119. 35512094 10.1073/pnas.2202439119 PMC9171807

[35] Ma J , Xia D. The use of blue native PAGE in the evaluation of membrane protein aggregation states for crystallization. J Appl Crystallogr 2008;41:1150–60. 19529836 10.1107/S0021889808033797 PMC2648668

[36] Otwinowski Z , Minor W. Processing of X-ray diffraction data collected in oscillation mode Methods Enzymol 1997;276:307–26. 27754618 10.1016/S0076-6879(97)76066-X

[37] Long F , Vagin AA, Young P, Murshudov GN. BALBES: a molecular-replacement pipeline. Acta Crystallogr D Biol Crystallogr 2008;64:125–32. 18094476 10.1107/S0907444907050172 PMC2394813

[38] Collaborative Computational Project, Number 4. The CCP4 suit: programs for protein crystallography. Acta Crystallogr D Biol Crystallogr 1994;50,760–3. 15299374 10.1107/S0907444994003112

[39] Adams PD , Grosse-Kunstleve RW, Hung LW, Ioerger TR, McCoy AJ, Moriarty NW, . PHENIX: building new software for automated crystallographic structure determination. Acta Crystallogr D Biol Crystallogr 2002;58:1948–54. 12393927 10.1107/s0907444902016657

[40] Murshudov GN , Vagin AA, Dodson EJ. Refinement of macromolecular structures by the maximum-likelihood method. Acta Crystallogr D Biol Crystallogr 1997;53:240–55. 15299926 10.1107/S0907444996012255

[41] Vagin A , Teplyakov A. MOLREP: an automated program for molecular replacement. J Appl Crystallogr 1997;30:1022–5.

[42] Emsley P , Cowtan K. Coot: model-building tools for molecular graphics. Acta Crystallogr D Biol Crystallogr 2004;60:2126–32. 15572765 10.1107/S0907444904019158

[43] Rossmann MG . The molecular replacement method. Acta Crystallogr A 1990;46:73–82. 2180438 10.1107/s0108767389009815

[44] Kobe B , Gleichmann T, Horne J, Jennings IG, Scotney PD, Teh T. Turn up the HEAT. Structure 1999;7:R91–7. 10378263 10.1016/s0969-2126(99)80060-4

[45] Liu X , Onda M, Watson N, Zhou Q, Hassan R, Ho M, . Highly active CAR T cells that bind to a juxta-membrane region of mesothelin and are not blocked by shed mesothelin. Proc Natl Acad Sci U S A 2022;119:e2202439119. 35512094 10.1073/pnas.2202439119 PMC9171807

[46] Shi X , Jarvis DL. Protein N-glycosylation in the baculovirus-insect cell system. Curr Drug Targets 2007;8:1116–25. 17979671 10.2174/138945007782151360 PMC3647355

[47] Jumper J , Evans R, Pritzel A, Green T, Figurnov M, Ronneberger O, . Highly accurate protein structure prediction with AlphaFold. Nature 2021;596:583–9. 34265844 10.1038/s41586-021-03819-2 PMC8371605

[48] Kobe B . Autoinhibition by an internal nuclear localization signal revealed by the crystal structure of mammalian importin alpha. Nat Struct Biol 1999;6:388–97. 10201409 10.1038/7625

[49] Huber AH , Nelson WJ, Weis WI. Three-dimensional structure of the armadillo repeat region of beta-catenin. Cell 1997;90:871–82. 9298899 10.1016/s0092-8674(00)80352-9

[50] Kucukelbir A , Sigworth FJ, Tagare HD. Quantifying the local resolution of cryo-EM density maps. Nat Methods 2014;11:63–5. 24213166 10.1038/nmeth.2727 PMC3903095

[51] Zhang YF , Phung Y, Gao W, Kawa S, Hassan R, Pastan I, . New high affinity monoclonal antibodies recognize non-overlapping epitopes on mesothelin for monitoring and treating mesothelioma. Sci Rep 2015;5:9928. 25996440 10.1038/srep09928 PMC4440525

[52] Iwahori K , Osaki T, Serada S, Fujimoto M, Suzuki H, Kishi Y, . Megakaryocyte potentiating factor as a tumor marker of malignant pleural mesothelioma: evaluation in comparison with mesothelin. Lung Cancer 2008;62:45–54. 18394747 10.1016/j.lungcan.2008.02.012

