# Supplementary material for: Structures of Cancer Antigen Mesothelin and Its Complexes with Therapeutic Antibodies
Source: Cancer Res Commun. 2023 Feb 1;3(2):175–91. doi: 10.1158/2767-9764.CRC-22-0306 (PMC10035497; doi:10.1158/2767-9764.CRC-22-0306)
Supplement: Table S1-S7 — Table S1: Plasmids and primers used for protein expression. Table S2: Characterization of MSLN expression constructs. Table S3: Structural alignments of MSLN models. Table S4: Lists of interactions. Table S5: Buried surface area for three MSLN-Fab complexes. Table S6: Observed saccharide attachments at predicted glycosylation sites. Table S7: Dali search result. [file crc-22-0306-s01.pdf]

Structures of Cancer Antigen Mesothelin and Its Complexes with Therapeutic Antibodies

Jingyu Zhan[a*], Dong Lin[a*], Nathan Watson[b], Lothar Esser[a], Wai Kwan Tang[a], Alex Zhang[a], Xiufen Liu[b], Raffit Hassan[c], Anne Gleinich[d], Asif Shajahan[d], Parastoo Azadi[d], Ira Pastan[b§], and Di Xia[a§]

[a]Laboratory of Cell Biology, [b]Laboratory of Molecular Biology, and [c]Thoracic and GI Malignancies Branch, Center for Cancer Research, National Cancer Institute, National Institutes of Health, Bethesda, MD, USA 20892

[d]Complex Carbohydrate Research Center, University of Georgia, Athens, GA 30602

Supplemental information

Supplemental tables

Table S1. Plasmids, restriction sites, and primers used in expressing full-length and fragments of human MSLN.

Construct	Vector	Restriction sites		Forward primer	Reverse primer
		1	2		
MSLN-132	pET42b	NdeI	HindIII	GGAATTCCATATGGAAGTGGAGAAGACAGCCTGTCCTTCAGGCAAGAAG	CCTGAAGCTTTCAATGATGATGATGATGATGGTCTAGCTGGCCCCTTCC
MSLN-168	pET24a	NdeI	HindIII	GGAATTCCATATGGAAGTGGAGAAGACAGCCTGTCCTTCAGGCAAGAAG	GTGAAGCTTTCAATGATGATGATGATGATGGGCCTGACCGCCCAG
MSLN-207	pET24a	NdeI	HindIII	GGAATTCCATATGGAAGTGGAGAAGACAGCCTGTCCTTCAGGCAAGAAG	GTGAAGCTTTCAATGATGATGATGATGATACCCAGGAAGGACTGGATC
MSLN-263	pET24a	NdeI	HindIII	GGAATTCCATATGGAAGTGGAGAAGACAGCCTGTCCTTCAGGCAAGAAG	GTGAAGCTTTCAATGATGATGATGATGATGGCGGTGCCGCTCCTC
FL-MSLN	pAcGP67A	SmaI	NotI	ATTACCCGGGGAAGTGGAGAAGACAGCC	ATTAAATTGCGGCCGCTTAGTGATGATGATGATGATGGCTGCTGCTGCCGCGCGGCACGAGGGCCTCTTGCAC

Table S2. Expression and characterization of various mesothelin constructs[a]

Construct	Residue range	No. of cysteines	Expressed form	M.W. (kDa)	Purified/ refolded	Structure obtained	Complex with Fab	Structure of complex	Ref
MSLN-64	302-359	2	soluble	6.7	Yes	Yes	Yes	Yes	24
MSLN-132	296-426	2	soluble	15.5	Yes	No[b]	Yes	No[b]	This work
MSLN-168	296-462	3	Incl. body	19.9	No	No	No	No	This work
MSLN-207	296-501	4	Incl. body	24.4	Yes	Yes	Yes	No[b]	This work
MSLN-263	296-557	4	Incl. body	30.5	No	No	No	No	This work
fl-MSLN	296-600	4	soluble	35.2	Yes	Yes	Yes	Yes	This work
C-term	582-598	0	synthetic	1.8	-	Yes	Yes	Yes	This work

a - all constructs were expressed in *E. coli* strain Rosetta (DE3), except for the fl-MSLN, which was expressed in insect cells.
b - low-resolution diffracting crystals obtained.

Table S3. Rms deviations (Å) of structural alignments for different constructs and domains of mesothelin[a].

	MSLN-207^{A} (299-427)	MSLN-207^{B} (299-427)	fl-MSLN /Fab[b] (299-585)	fl-MSLN-245^{A} (299-543)	fl-MSLN-245^{B} (299-546)	fl-MSLN-245^{C} (299-546)	fl-MSLN-245^{D} (299-546)	fl-MSLN-245^{E} (299-546)	fl-MSLN-245^{F} (299-546)
**MSLN-64 (299-359)**	0.45	0.45	0.39	0.49	0.50	0.49	0.50	0.50	0.49
**MSLN-207^{A} (299-427)**	-	0.77	1.28	1.25	1.25	1.32	1.52	1.46	1.45
**MSLN-207^{B} (299-427)**		-	1.52	1.33	1.37	1.41	1.59	1.54	1.59
**fl-MSLN/Fab (299-585)**			-	1.16	1.19	1.23	1.51	1.40	1.45
**fl-MSLN-245^{A} (299-543)**			0.39 (299-427)	-	0.24	0.21	0.62	0.61	0.56
**fl-MSLN-245^{B} (299-546)**			0.39 (299-427)		-	0.23	0.71	0.54	0.75
**fl-MSLN-245^{C} (299-546)**			0.40 (299-427)			-	0.67	0.52	0.74
**fl-MSLN-245^{D} (299-546)**			0.71 (299-427)				-	0.37	0.46
**fl-MSLN-245^{E} (299-546)**			0.72 (299-427)					-	0.49
**fl-MSLN-245^{F} (299-546)**			0.71 (299-427)						-

a - The Rms deviations were calculated by aligning the Cα atoms of the two structure coordinates using Pymol.
b - based on the fl-MSLN/Fab(MORAb) structure

Table S4A. Interactions between MSLN and MORAb-009[a]

Res. name	Atom type	Domain	Interactions Type	Distance (Å)	Chain/Res.	Atom type
K306	NZ	A	H-bond[b]	3.54	L/S31	OG
K306	NZ	A	H-bond	3.24	L/S92	OG
F317	CZ	A	Ar-Ar[c]	3.87	L/H94	CD2
F317	CE	A	Ar-Ar	3.52	L/W91	CE
F317	CZ	A	Hydrophobic	3.44	L/L95	CZ
K319	NZ	A	Charged	2.89	L/D50	OD1
W321	NE2	A	H-bond	3.29	L/Y32	OH
N340	O	A	H-bond	2.53	H/N55	ND2
N340	OD1	A	H-bond	3.55	H/N55	ND2
P343	CA	A	VDW	3.72	H/T52	CG2
P343	CB	A	VDW	3.65	H/L50	CD1
F344	O	A	H-bond	2.75	H/Y54	OH
T345	OG1	A	H-bond	2.46	H/G31	O
Y346	N	A	H-bond	3.36	H/G31	O
Y346	CE1	A	Ar-Ar	3.95	H/Y54	CG
Y346	CD2	A	Dipolar	3.09	H/G31	CA
Y346	CD2	A	H-bond	3.82	H/T30	OG1
E347	OE1	A	H-bond	3.33	H/D102	N
L349	CD1	A	VDW	3.27	H/Y54	OH
Y374	CE2	B	Dipolar	3.20	H/Y54	O
Y374	CB	B	VDW	4.00	H/Y54	CE2

a - Distances < 4 Å are used; b - H-bond, Hydrogen bond; c - Ar-Ar, Aromatic-aromatic interaction.

Table S4B. Interactions of residues between neighboring ARM and/or HEAT units in MSLN.

Repeat 1	Residue	Distance (Å)	Repeat 2	Residue	Repeat pairs	Construct end
ARM-1	None		ARM-2	None	ARM-1 vs. ARM-2	MSLN-64
ARM-2	F376	3.74	HEAT-3	L414	ARM-2 vs. HEAT-3	
ARM-2	F376	3.90	HEAT-3	V411		
ARM-2	F376	3.73	HEAT-3	L398		
HEAT-3	L399	3.71	ARM-4	F436	HEAT-3 vs. ARM-4	MSLN-132
HEAT-3	L399	3.94	ARM-4	L433		
HEAT-3	L419	3.57	ARM-4	A459		
ARM-4	L449	3.41	ARM-5	L476	ARM-4 vs. ARM-5	MSLN-168
ARM-4	C442	2.05	ARM-5	C468	S-S bond	
ARM-4	L441	4.00	ARM-5	V460		
ARM-4	L430	3.57	ARM-5	A459		
ARM-5	None		HEAT-6	None	ARM-5 vs. HEAT-6	MSLN-207
HEAT-6	L508	3.23	ARM-7	L526	HEAT-6 vs. ARM-7	MSLN-263
HEAT-6	L508	3.46	ARM-7	L543		
HEAT-6	L511	3.89	ARM-7	L542		
HEAT-7	F493	3.94	ARM-7	M518		

Table S4C. Interactions of Mab 15B6 with the C-terminal peptide[a]

Peptide residue	HC residue	Distance (Å)	Peptide residue	LC residue	Distance (Å)
Y586	T30	2.63	S292	N36	2.80
D590	R50	2.69	E595	Y35	2.82
S592	L100	2.99	A596	N54	2.78
			L597	N54	2.92

a - only distances < 3 Å are given.

Table S5. Buried surface area (BSA, Å^2) for the MSLN-64/Fab, fl-MSLN/Fab(MORAb) and C-term/Fab(15B6) complexes						
	Bound Mab	SC[a]	Total BSA[b]	BSA for MSLN/peptide	BSA for H chain	BSA for L chain
fl-MSLN/Fab	MORAb-009	0.68	1,918	1000	616	302
MSLN-64/Fab	MORAb-009	0.71	1,714	921	496	297
C-terminal peptide	15B6	0.81	1,528	845	336	347

a - SC refers to shape complementarity, 1.00 is perfect complementation.
b - Total BSA refers to BSA for both antigen and antibody. The buried surface area was calculated using the Areaimol in CCP4 .

Table S6. Observed saccharide attachments at predicted glycosylation sites

Chain	N388	N488	N515
fl-MSLN/Fab(MORAb)	GlcNAc-GlcNAc	No	No
fl-MSLN-245-A	GlcNAc	No	No
fl-MSLN-245-B	GlcNAc	No	No
fl-MSLN-245-C	GlcNAc	No	No
fl-MSLN-245-D	No	No	GlcNAc-GlcNAc
fl-MSLN-245-E	No	GlcNAc	No
fl-MSLN-245-F	No	No	GlcNAc-GlcNAc-BMA

Table S7. Dali search result

Rank	PDB-Chain	Z score	Rmsd (Å)	No. aligned	No. residues	% Identity	Description
1	4F3F-C	12.6	0.5	59	59	100	MORAb-009 with MSLN-64
2	4C0Q-B	5.8	12.7	215	749	9	Transporting-3 in complex with GTPase Ran
3	2XWU-B	5.2	5.0	184	918	11	Importin-13/UBC9 complex
4	6RE2-3	5.2	4.5	143	245	6	ASA-10: POLYTOMELLA F-ATP SYNTHASE
5	6RE3-3	5.2	4.5	143	245	6	ASA-10: POLYTOMELLA F-ATP SYNTHASE
6	6RDC-3	5.2	4.5	138	245	8	ASA-10: POLYTOMELLA F-ATP SYNTHASE
7	7CUN-G	5.1	10.9	151	895	9	INTEGRATOR COMPLEX SUBUNIT 1
8	1Z3H-B	5.1	10.5	164	914	9	IMPORTIN ALPHA RE-EXPORTER
9	3CIA-A	5.1	3.4	110	587	11	COLD-ACTIVE AMINOPEPTIDASE
10	6RE6-3	5.0	4.5	141	245	6	ASA-10: POLYTOMELLA F-ATP
