# Supplementary material for: Structures of Cancer Antigen Mesothelin and Its Complexes with Therapeutic Antibodies
Source: Cancer Res Commun. 2023 Feb 1;3(2):175–91. doi: 10.1158/2767-9764.CRC-22-0306 (PMC10035497; doi:10.1158/2767-9764.CRC-22-0306)
Supplement: Figure S1 — Arrangement of MSLN molecules in the crystal. [file crc-22-0306-s02.pdf]

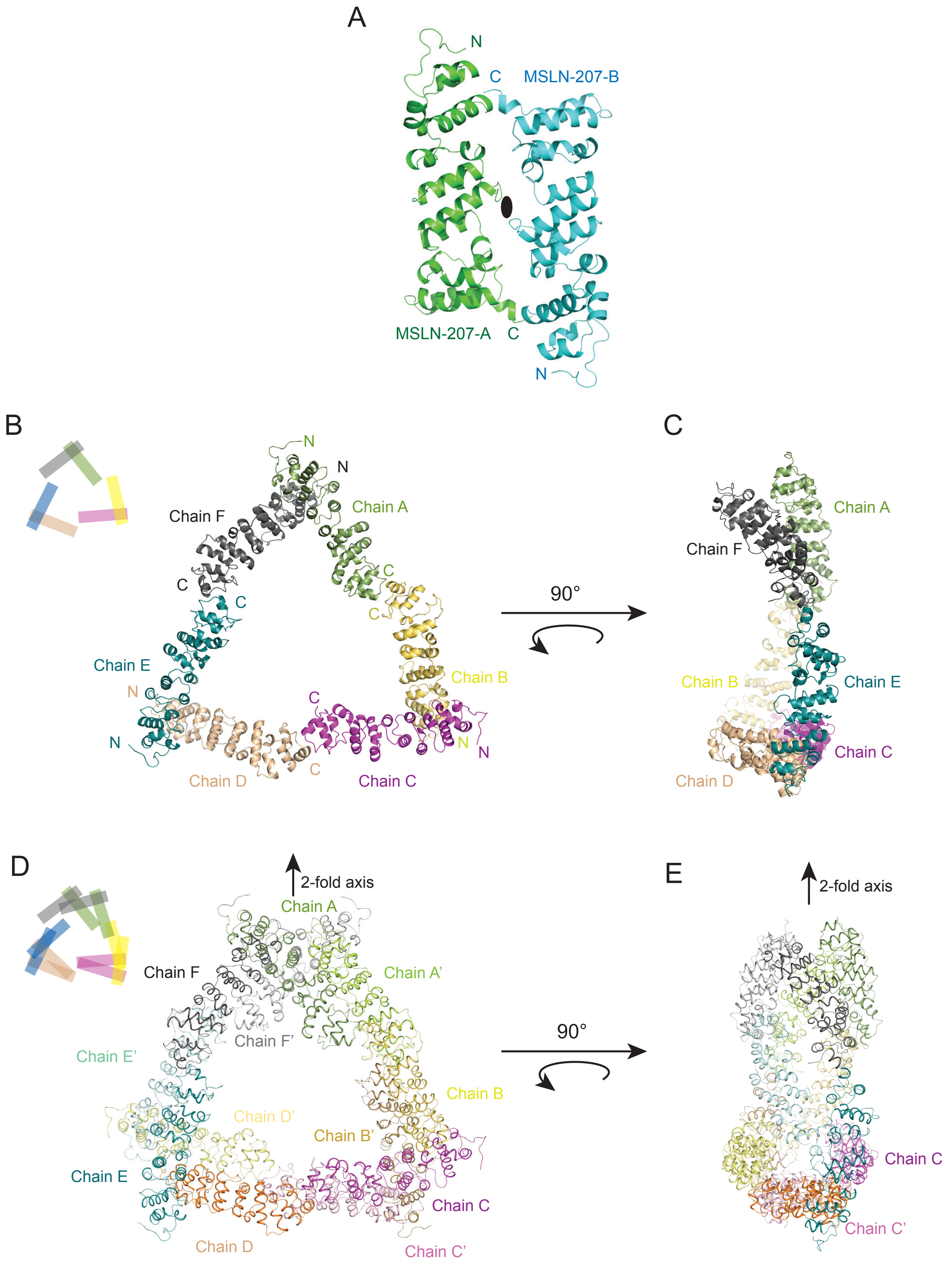

Zhan et al., Figure S1

Figure S1. Arrangement of MSLN molecules in the crystal. (A) Dimeric association of the two MSLN-207 molecules. Structures of the two MSLN-207 chains in a crystallographic AU are rendered as cartoon diagrams with chains A and B in green and cyan, respectively. Both N- and C-termini are labeled. Also shown is a black oval indicating the position of a non-crystallographic 2-fold symmetry axis. (B) Arrangement of the six truncated MSLN model chains (fl-MSLN-245) form a hexameric triangle in the crystallographic AU of fl-MSLN crystallized in the symmetry of *C*2 space group. The triangular arrangement is a result of an apparent pseudo three-fold symmetry. Each chain is assigned a unique color and labeled. The small diagram at top-left corner shows the arrangement in a cartoon fashion. (C) View of a 90° rotation of (B). (D) Two hexameric triangles related by a 2-fold symmetry axis shown as a vertical arrow form the basic motif of the crystal. (E) A 90° rotated view of (D).
