# Supplementary material for: Structures of Cancer Antigen Mesothelin and Its Complexes with Therapeutic Antibodies
Source: Cancer Res Commun. 2023 Feb 1;3(2):175–91. doi: 10.1158/2767-9764.CRC-22-0306 (PMC10035497; doi:10.1158/2767-9764.CRC-22-0306)
Supplement: Figure S2 — Comparison of experimental MSLN structure with those of computational models. [file crc-22-0306-s03.pdf]

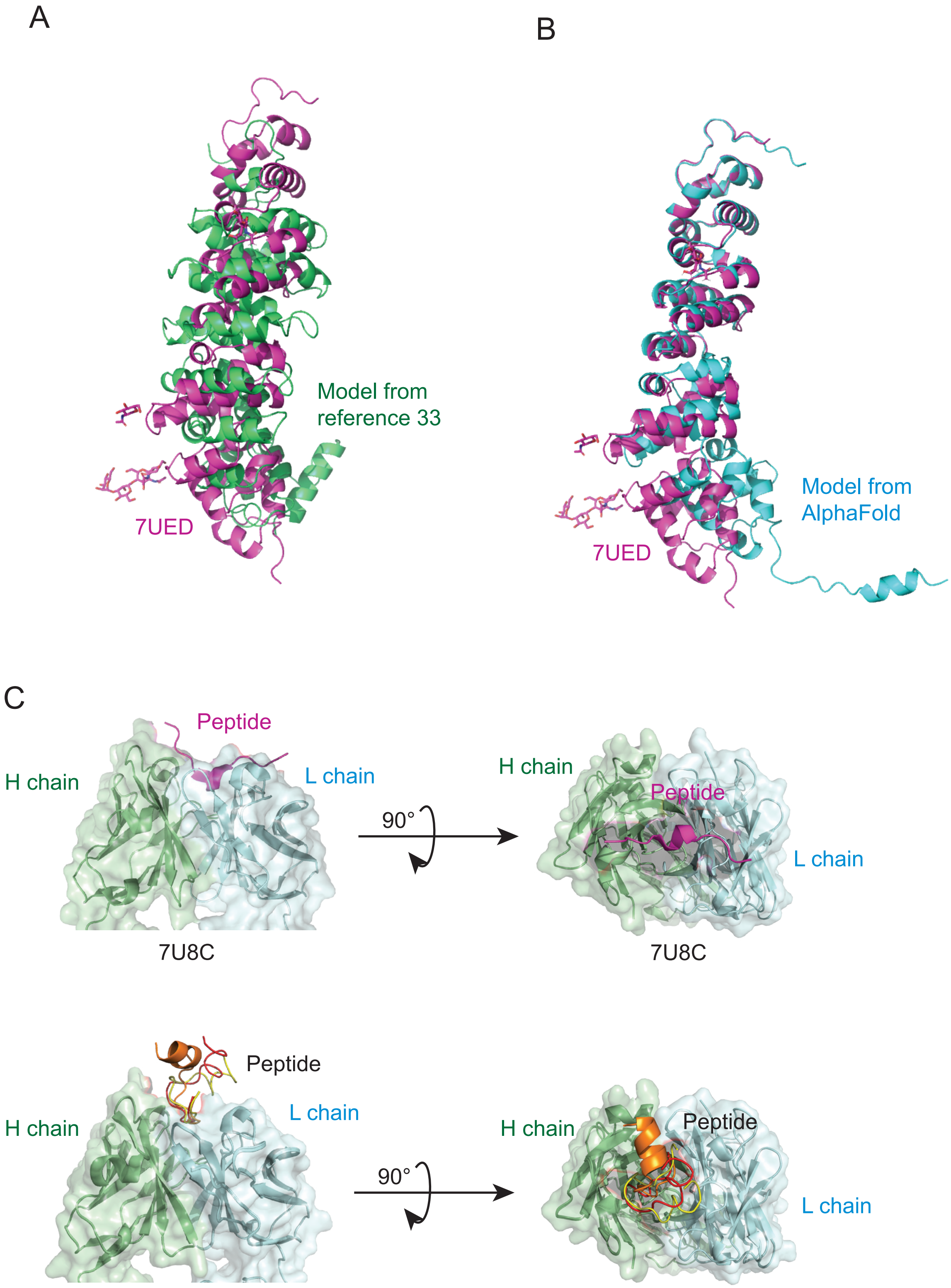

Zhan et al., Figure S2

Figure S2. Comparison of experimental MSLN structure with those of computational models. (A) Structure superposition between experimental MSLN model (PDB: 7UED, magenta) and a published computational model (33) (green) with a rms deviation of 15.8 Å. Both structures are rendered as cartoon models. (B) Structure superposition between 7UED (Magenta) and model generated by AlphaFold2 (Cyan), which gave rise to a rms deviation of 3.0. Here the N-terminal 64 residue fragment is aligned with a rms deviation of 0.4 Å, indicating that AlphaFold2 incorporates available information from the PDB database. (C) Comparison of the structure of C-terminal peptide/Mab(15B6) complex (PDB:7U8C) with models predicted by AlphaFold-Multimer. The top two panels show the structure of C-term/Fab of 15B6 complex (PDB:7U8C), as a cartoon representation, in two orthogonal orientations. The peptide is bound across a grove made of both heavy (H, green) and light (L, cyan) chains. The bottom two panels are the predicted models, in two orientations, for the bound peptide. All predicted models put the peptide between the heavy and light chains. The peptide models have an average rms deviation of 4.0 Å when aligned to the experimental one for all residues.
