# Supplementary material for: Structures of Cancer Antigen Mesothelin and Its Complexes with Therapeutic Antibodies
Source: Cancer Res Commun. 2023 Feb 1;3(2):175–91. doi: 10.1158/2767-9764.CRC-22-0306 (PMC10035497; doi:10.1158/2767-9764.CRC-22-0306)
Supplement: Figure S3 — Mapping epitopes of monoclonal antibodies to the structure of MSLN. [file crc-22-0306-s04.pdf]

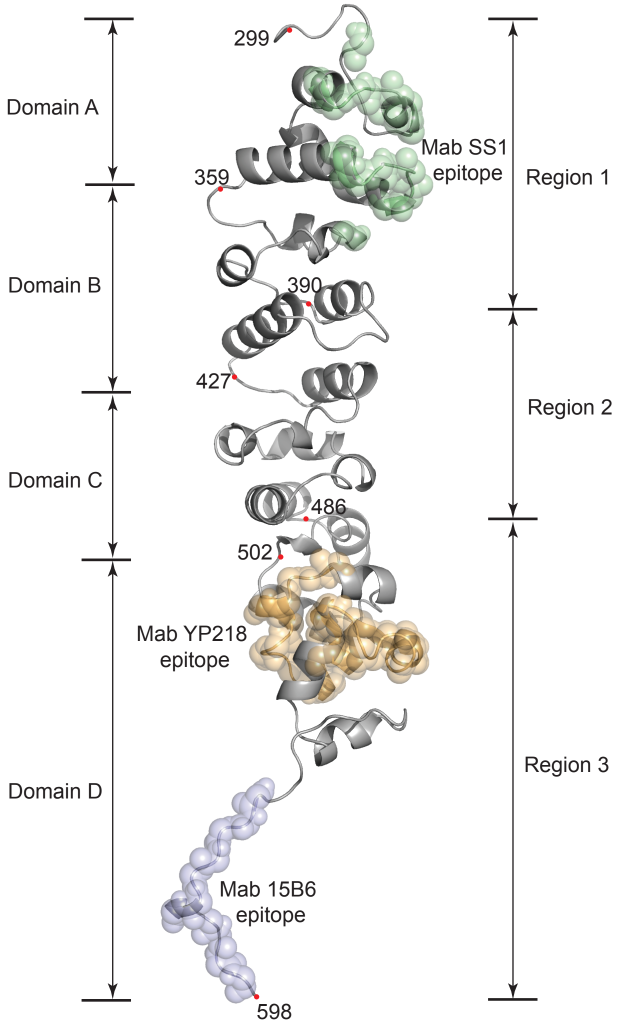

Zhan et al., Figure S3

Figure S3. Mapping epitopes of monoclonal antibodies to the structure of MSLN. fl-MSLN is subdivided into four sub-domains (Domains A, B, C and D) based on structural superposition. Domains A, B and C are stable, while Domain D is relatively mobile. In a previous study (42), sequence of MSLN was divided into three regions (Regions 1, 2, and 3). Among 232 isolated high-affinity monoclonal antibodies, 223 mapped their epitopes to Region 1, 5 to Region 2, and 3 to Region 3. MORAb-009 or SS1 is a typical Region 1 antibody with an epitope, shown as green mainchain surface, contributed by nonconsecutive sequence fragments of MSLN. 15B5 targets a C-terminal linear epitope, shown as purple mainchain surface. YP218 also targets Region 3 and is predicted to have the epitope shown as brown surface.
